# Population Morphology Implies a Common Developmental Blueprint for *Drosophila* Motion Detectors

**DOI:** 10.1101/2025.11.15.688637

**Published:** 2025-12-21

**Authors:** Nikolas Drummond, Arthur Zhao, Alexander Borst

**Affiliations:** 1 Department of Circuits - Computation - Models, Max Planck Institute for Biological Intelligence, Munich, Germany; 2 Reiser Lab, HHMI Janelia Research Campus, Virginia, USA

## Abstract

Detailed characterisation of neuronal morphology provides vital clues for understanding the wiring logic and development of neural circuits. As neuron arbours can both span large distances, and be densely interwoven, one major challenge is acquiring and quantifying fine structures for independent arbours over large spatial extents. Recent whole-brain electron microscopy (EM) connectomes of *Drosophila melanogaster* provide an ideal opportunity to study fly neuronal morphology at scale. Utilising this rich resource, we developed novel computational methods and morphological metrics to perform the most comprehensive morphological analysis of the dendrites of T4 and T5 neurons in the fly brain.

T4 and T5 neurons are the first direction-selective neurons in the visual pathway. They are the most numerous cell types in the fly brain (∼ 6000 within each optic lobe) and as a population, their compact dendritic arbours span the entire visual field. They are classified into four subtypes (a, b, c, and d). Each subtype encodes one of four orthogonal motion directions (up, down, forwards, backwards). The dendrites of these neurons form in two distinct neuropils, the Medulla (T4) and the Lobula (T5), and respond to ON (light increments, T4) and OFF (light decrements, T5) motions. T4 and T5 neurons’ dendrites are oriented against their preferred direction of motion. However, the differences beyond their characteristic orientation, both between T4 and T5, as well as within subtypes, have remained poorly understood.

Our analysis reveals a high degree of structural similarity between T4 and T5, and within their subtypes. Particularly, the geometry of branching, section orientation, and tree-graph structure of these dendrites show only minor variability, with no consistent separation between T4 and T5, or their subtypes.

These results indicate that, despite forming in different neuropils, and serving distinct motion directions, T4 and T5 dendrites follow closely aligned morphological patterns. This suggests a shared developmental mechanism.

## Author summary

With *nm*-resolution neuron morphologies available from recent electron microscopy-based reconstructions, we are able to extract and analyse the dendrites of all T4 and T5 neurons within the right optic lobe of a single adult Drosophila. Investigating elements of spatial embedding, geometry, and their graph-theoretical structure, we show brain region specific variability in the spatial occupancy of these dendrites, but high structural stereotypy. From these analyses, we conclude that although these dendrites originate in differing brain regions, with differing input partners, and perform differing, subtype-specific, detection of motion directions, the underlying developmental process is most likely shared across both T4 and T5 and their subtypes.

## Introduction

The importance of neuron morphology has been recognised within neuroscience for over a century [1–3]. The specific morphology of a neuron determines its role in networked computations through its connectivity with input and output neuron partners [4]. Further, the precise branching structure is intrinsically important to the electrophysiological properties of the neuron, and plays an important role in the computations performed on the inputs to the neuron [5–8]. The quantification of neuron morphology additionally sheds light on neuron development, as well as the relationship between structure and function [2, 9].

**Fig 1.**
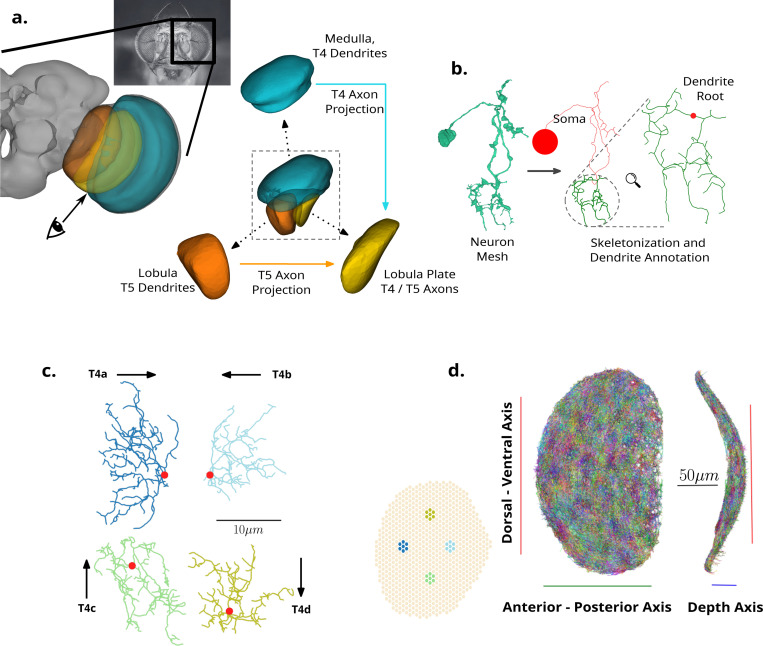
Overview of *Drosophila* optic lobe and T4 / T5 dendrites a) (*left*) View of the right Optic lobe available in flywire, with Medulla (Turquoise), Lobula (Orange) and Lobula Plate (Yellow) shown. (*right*) Exploded view of neuropils coloured from perspective illustrated by the eye schematic in *left*. Coloured arrows denote axonal projections for T4 (Medulla to Lobula Plate) and T5 (Lobula to Lobula Plate). b) Illustration of skeletonization and Dendrite annotation procedure from mesh to extracted example dendrite. c) Example extracted T4 dendrites for each subtype. Red points show dendrite root points (first branching point). Arrows show encoded directional tuning of each subtype, inverse to the direction of dendrite outgrowth. d) All extracted T4 dendrites in the globally aligned Dorsal-Ventral (DV) / Anterior-Posterior (AP) view (*left*) and DV / Depth view (*right*). Insert shows visual column map available in FLYWIRE. Coloured hexagons illustrate Dorsal, Ventral, Anterior, and Posterior example columns used for alignment. Each example dendrite in c matches by colour the T4 dendrite assigned to the centre of each group of coloured hexagons.

T4 and T5 neurons are the first motion direction-selective neurons within the *Drosophila* visual system. They process information across three retinotopically arranged neuropils, the Medulla, Lobula, and Lobula Plate (Fig. 1,a). T4 neurons respond preferentially to moving light increments (ON motion), receiving their inputs in the Medulla and outputting in the Lobula Plate. T5 neurons respond to moving light decrements (OFF motion), receiving inputs within the Lobula and again outputting within the Lobula Plate. T4 dendrites innervate Medulla layer 10, whereas T5 dendrites innervate Lobula layer 1. Each T4/T5 dendrite forms synapses with the same set of neuropil-specific input partners, in a distinct spatial order, clearly distinguishing the ON and OFF pathways [10].

How T4 and T5 dendrite morphology may differ between the ON and OFF pathways, if at all, is poorly understood. Their existence in two separate neuropils, and differing input partners raise the question of how their local environment impacts morphology. However, both share similar functional and biophysical properties [11]. The necessity of spatial integration of information across the visual field in order to facilitate motion detection implies a highly controlled mechanism [11, 12]. If this leads to morphological differences between the two pathways, whilst maintaining function, remains to be seen.

Both the Medulla and Lobula possess a retinotopic mapping through their columnar structure (Fig. 1,d, insert), and each T4/T5 dendrite spans across approximately seven adjacent columns [10, 11]. Both T4 and T5 cells form four subtypes (a, b, c, d), which encode the four cardinal directions of body motion (forwards, backwards, downwards, upwards) [13]. Strikingly, each dendrite subtype is physically oriented in a direction inverse to its preferred functional direction (Fig. 1,c), determined by the structure of the compound eye [14]. This orientation is dominant within the central region of the eye. Additionally, evidence in favour of a further functional subtype division along the horizontal axis (a and b subtypes) is given in [15]. The subtype-specific spatial asymmetry within these dendrites facilitates the integration of information from adjacent points in visual space which is necessary for motion detection [11, 12].

However, beyond this characteristic orientation, it is unclear if dendrite morphology differs between subtypes. The necessity of dendrites to be oriented along a specific visual axis may require differing morphologies for each subtype, or along each axis, grouping horizontal (a, b) and vertical (c, d) subtypes in a manner whereby each pair is a mirrored copy. Alternatively, a solution may be present which allows for shared morphology to asymmetrically span the required region of columnar space independent of subtype. Further, it is not clear at what level of morphological description this asymmetry emerges. At one extreme, this asymmetry could be a result of differing spatial occupancy between subtypes. On the other, the fundamental geometry or topology of each dendrite could differ in a manner that facilitates their directionality.

The developmental origins of T4 and T5 neurons preceding the emergence of dendritic orientation are also well understood. All T4 and T5 neurons form from the same neuroblasts, with subtype specificity being determined during development prior to innervation into their target neuropils [16, 17]. Each individual neuroblast will form into four T4 and T5 neurons. Specifically, a single neuroblast (NB) will form into either a set of T4a, T4b, T5a, and T5b neurons; or T4c, T4d, T5c, and T5d. During this developmental cascade, the final type division during development separates T4 and T5 neurons of the same subtype, for example, T4a and T5a. Neurons from the same neuroblast innervate the Medulla and Lobula along a temporo-spatial gradient [16], indicating that neuron pairs from the same neuroblast innervate similar regions of the visual field.

It is apparent from this that all T4 and T5 neurons share a common developmental origin. It is not clear, however, if the emergence of orientation, and manner by which these dendrites occupy space, differs between the subtypes and as such is a result of differing developmental programmes. All dendrites will occupy their initial starting positions within the Medulla and Lobula, and have there ultimate subtype determined through their NB progenitor, prior to the emergence of there dendritic orientation [16, 17]. Through investigating the quantitative differences in the structure of these dendrites, we aim to determine if the characteristic orientation of these dendrites is likely a result of differing developmental programmes, or a result of a shared algorithm across subtypes, independent of their eventual orientation.

With the release of a fully reconstructed connectome of an adult female Drosophila brain (flywire) [18–20], alongside a full inventory of the optic lobe [21], the wealth of morphological data within *Drosophila* is unparalleled. By isolating the dendrites of all T4 and T5 neurons within a single optic lobe we are able to perform in-depth analysis of their morphology in order to identify the extent of similarities and differences. Doing so allows us to better understand the important features determining their characteristic asymmetry and developmental algorithms.

We separate our analysis of T4 and T5 dendrite morphology into three components. First, we examine the spatial embedding of these dendrites within their densely overlapping environment. In doing so we identify neuropil-specific variability (T4 vs T5) in the spatial distribution of dendrite size whereby T4s seemingly mirror spatial lens size variability in a manner not present in T5 [22–24]. Second, we consider the orientation and geometry of individual dendrites. Here we find that 3D bifurcation geometry and section orientation relative to the start of dendritic branching is conserved across both types and subtypes, indicating that the arbourisation structure, and by extension the manner by which these dendrites fill space, is shared regardless of neuropil or their characteristic asymmetry. Finally, we examine the topological and graphical structure of T4 and T5 dendrites. Here, again, we find no systematic differences between T4 and T5, or their subtypes indicating a shared dendritic structure.

These results are in favour of a shared developmental algorithm across both T4 and T5. Additionally, we can pinpoint the origin of their characteristic asymmetry to be at the level of the orientation in global space of individual dendritic arbours, and not a result of differing spatial occupancy, geometry, or topology.

## Results

### Note on Statistical Analysis and Outcomes.

Due to the large n available within this dataset, standard practices relying on reporting p-values are not suitable here. This is due to the fact that with large datasets extremely small effects can produce very small p-values, which limits the interpretability of significance testing alone [25]. As such, we focus on reporting effect sizes in order to emphasise the magnitude of differences over statistical significance alone [26, 27]. In order to better facilitate the interpretability of results, we briefly detail the statistical methods and reported metrics here.

We analysed group differences using ordinary least squares (OLS) regression models. Main effects of type, subtype, and their interaction (denoted as type:subtype) were evaluated with analysis of variance (ANOVA). When continuous covariates were included, we used analysis of covariance (ANCOVA) to account for their contribution.

For ANOVA and ANCOVA models, we report partial eta squared, $\eta_{p}^{2}$, which quantifies the proportion of variance uniquely attributed to each factor given the model structure [27] (See Materials and Methods, Statistical Analysis). Guidelines from [28] are used for interpretation: ”small” effect size as ≥ 0.01, a ”medium” effect size as ≥ 0.06, and a ”large” effect size as ≥ 0.14. These thresholds relate to 1%, 6% and 14% of the variance within the model being explained by a factor. For *post hoc* pairwise comparisons, we report Cohen′s_*d*_ (”small”: ≥ ±0.2, ”medium”: ≥ ±0.5, ”large”: ≥ ±0.8) [28]. As Cohen′s_*d*_ is signed, it provides the direction of difference between groups. We additionally report slopes from OLS regression models (β), and differences between slopes (Δβ) where applicable when considering main effects of continuous predictors and their interactions.

In order to quantify uncertainty around effect sizes we additionally compute non-parametric bootstrap confidence intervals (1000 bootstrap replicates). An effect is considered meaningfully large when both the point estimate and its entire confidence interval exceed the relevant threshold for a small effect size.

### T4 and T5 Dendrite Annotation and Pre-processing.

The flywire dataset comprises of ∼ 140000 fully reconstructed neurons from a single 4 × 4 × 40*nm* resolution electron microscopy (EM) image stack [18–20, 29]. Neuron type annotations within the right optic lobe of this dataset are available through [21], and include ∼ 6000 annotated T4 and T5 neurons. Each neuron is represented as a 3D surface mesh which, in order to facilitate the analysis of morphology, is modelled as a tree-graph (See Materials and Methods, T4 and T5 Neuron Morphologies; Neurons as Tree Graphs). We further require the accurate annotation of the dendritic compartment of each neuron tree-graph in order to extract and analyse dendrite morphology.

Previously, separation of neuron morphologies into biologically relevant subtrees, such as the axon and dendrite, has been done manually [30] or derived from synapse data [31, 32]. Within this dataset of ∼ 6000 neurons, manual annotation of each dendrite is not practical. Methods based on the input-to-output ratios of synapses [31, 32] assume clearly defined information flow between compartments, which does not always hold [30], and require additional synapse annotations. We instead implement a novel, semi-automated method to annotate the dendritic compartment of T4 and T5 neurons based on tree structure alone.

Skeletonization, the method of extracting tree-graph representations from neuron surface meshes, is implemented using a standard procedure [33, 34]. The correct rooting of each neuron at the soma is then manually verified and corrected if needed. Given a directed tree-graph we define a subtree as all nodes and edges downstream of a specified node. Given a correctly rooted neuron skeleton we find the branch node which minimises the total cable length within the subtree, and maximises the number of terminal, or leaf, nodes (See Materials and Methods, Dendrite Extraction and Data Pre-processing, Fig. 1,b). A second manual verification of all neurons is then completed in order to ensure that the annotated subtree defined by the identified node isolates the complete dendrite. During this second manual review step, the reviewer can additionally fix individual errors in the case where the dendrite subtree is clearly defined by a single node. Both manual review steps are completed using a custom-built GUI. This annotation and review can be completed for ∼ 6000 neurons in a short time by a single expert reviewer. Using this pipeline we isolate ∼ 95% of available dendrites for all T4 and T5 neurons within the right optic lobe (*n* = 5828). Supplementary table 1 provides counts and proportions of retained dendrites for analysis in comparison to all available T4 and T5 neurons by subtype.

As T4 and T5 dendrites exist in two separate neuropils (Fig. 1,a) we also align both sets of dendrites to a common reference space (Fig. 1,d, Materials and Methods, Alignment and Scaling). In brief, taking advantage of the fact that both Medulla layer 10 and Lobula layer 1 are well approximated by a section on the surface of a sphere (Fig. 1,d), we compute the spherical fit and centre each T4 and T5 dendrite population to their respective sphere. Each population is then rotated and translated in order to ensure their principal axis align with the polar axis and equatorial plane. Finally, we ensure that the anatomically dorsal population for both T4 and T5 is positioned within the northern hemisphere to ensure that both T4 and T5 dendrite populations are aligned with the same Dorsal-Ventral (DV) and Anterior Posterior (AP) space. This final rotation is completed with reference to the column annotations within flywire (Fig. 1,d insert). These available annotations assign neurons to a column in visual space, but do not include neuropil-specific spatial columns, necessitating this final step in order to facilitate the shared DV/AP alignment of the T4 and T5 dendrite populations.

### Dendrite Spatial Embedding

We first turn to understanding the spatial structure and embedding of T4 and T5 dendrites in order to understand differences in the manner in which these dendrites occupy their densely overlapping environment. Here we focus on the shape and volume of the spanned region of T4 and T5 dendrites, the manner in which these dendrites innervate across the depth axis of their respective neuropil layers, and the spatial relationships between the first branching point of different dendrites.

#### Dendrite Shape

**Fig 2.**
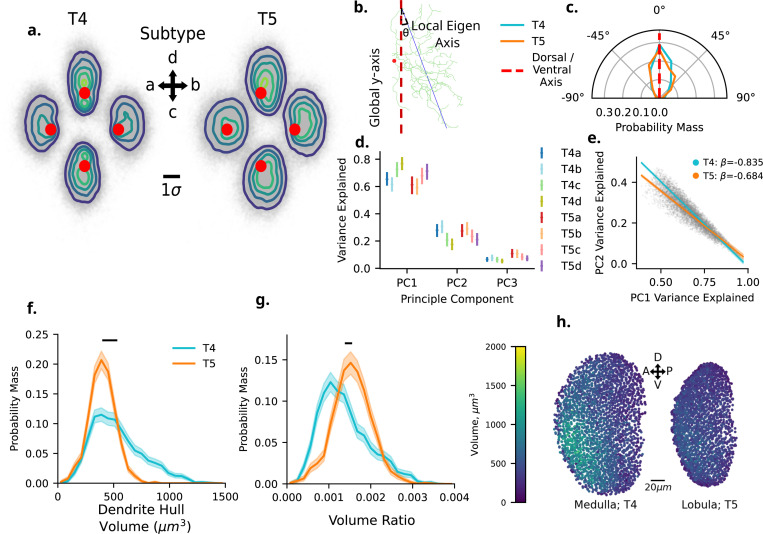
Spatial Embedding and Volume of T4 and T5 Dendrites. a) Scaled and locally aligned subtype specific dendrite populations. Each individual dendrite is aligned to its internal principal axis and scaled within each type specific population (T4 and T5) to one standard deviation of the population dispersion along the first principal axis. Individual dendrites are centred on the root of the dendritic tree (red points). KDE’s show point density for each subtype. b) Schematic illustrating angular deviation between a dendrites internal first principal axis (blue line) and the global Dorsal-Ventral (DV) axis (red dashed line). Red point shows the dendrite root. c) Distribution of angles between DV axis and dendrites first principal axis for T4 and T5 dendrites d) Variance explained along each principle component (PC) for each subtype. e) Relationship between variance explained between first and second principle components for T4 and T5 dendrites. f) Distribution of raw convex hull volumes for T4 and T5 dendrites. Black horizontal lines denotes difference in means. g) Distribution of convex hull volumes scaled by Medulla layer 10 volume (T4) and Lobula layer 1 volume (T5). Black horizontal lines denotes difference in means. h) Spatial distribution of T4 and T5 convex hull volumes within their respective neuropil (T4, Medulla, left; T5, Lobula, right) on the globally aligned DV / AP plane.

T4 and T5 dendrites are often characterised by their asymmetrical orientation [13], whereby the direction of dendrite outgrowth is inverse to the direction of functionally encoded motion (Fig. 1,c). In order to visualise the occupied space of dendrites by subtypes we align individual dendrites to their internal principal axis, ensuring that the major axis aligns with the y-axis, and scale by the population standard deviation along the y-axis within type (See Materials and Methods, Alignment and Scaling). By centring individual dendrites on the first branch point, we can overlay all dendrites within subtypes (Fig. 2,a). This shows us a clear elliptical spatial occupancy shared across all dendrite subtypes. Having centred dendrite subtype populations on their initial branching point, we also observe clear offsets from the centre of each elliptical shape for each subtype (Fig. 2,a; Red points), with the exception of T5a subtypes, where the root position appears more centrally positioned.

Given these dendrites’ characteristic asymmetry, it is unclear from this alone if the internal principal axis, and the extension of these dendrites in space, is shared along either the dorsal-ventral (DV) or anterior-posterior (AP) axis across all subtypes, or differs between horizontally oriented (a, b) and vertically oriented (c, d) subtypes. In order to investigate this we measure the angle between each dendrites’ internal principal axis and the global DV axis (Fig. 2,b). We find that the internal principal axis of all types and subtypes is aligned well with the global DV axis (Fig. 2,b). There is no meaningfully large difference between Types $\left(\eta_{p}^{2}\leq 0.01\right)$. We find a small main effect of Subtypes ($\eta_{p}^{2}=0.028$, CI : [0.020, 0.038]). *post hoc* pairwise comparisons show some variability between subtypes, although all observed effect sizes are small to medium (0.2 ≤ Cohen′s_*d*_ ≤ 0.8).

Specifically, we find a small effect size showing a>c (Cohen′s_*d*_ = 0.371, CI : [0.299, 0.448]), b>c (Cohen′s_*d*_ = 0.422, CI : [0.346, 0.494]). We find a medium effect size showing d>c (Cohen′s_*d*_ = 0.557, CI : [0.481, 0.633]). These results indicate that the vertical (c, d) subtypes deviate more around the DV axis in comparison to the horizontal (a, b) subtypes. However, the mean difference between the largest effect size (d>c) corresponds to a mean difference of only 13.828°, indicating that this variability is still aligned with the DV axis. The interaction term investigating if the pattern differs between subtypes within types is not meaningfully large $\left(\eta_{p}^{2}\leq 0.01\right)$.

In order to understand if these dendrites differ in their spread along the DV, AP, and depth axis we examine the variance explained along each principal component (PC, Fig. 2,d). Our analysis is conducted within each PC separately. As we have shown that PC1 is aligned well with the DV axis, it follows that PC2 is aligned well with the AP axis, and PC3 aligns well with the depth of each dendrite’s respective neuropil layer.

Within PC1 we find no meaningfully large difference between types $\eta_{p}^{2}\leq 0.01$. Between subtypes we find a large effect ($\eta_{p}^{2}=0.185$, CI : [0.165, 0.208]). *Post hoc* pairwise comparisons find all comparisons with the exception of a vs. b subtypes are meaningfully large (Cohen′s_*d*_) > 0.2. Considering the mean differences between subtypes, there is a clear structuring in this result. The variance explained along the first axis, which translates to the DV axis, is ordered in a subtype-specific manner following d>c>b>a.

Within PC2 we again find no meaningfully large difference between types $\left(\eta_{p}^{2}\leq 0.01\right)$. We again observe a large effect size between subtypes ($\eta_{p}^{2}=0.183$, CI : [0.164, 0.206]). Again, with the exception of a vs. b subtypes, *post hoc* comparisons all show meaningfully large effect sizes Cohen^*′*^s_*d*_ > 0.2. Interestingly however, the previously observed ordering between subtype means is reversed (d>c>a>b). Interestingly, we find a small main effect for the Type:Subtype interaction ($\eta_{p}^{2}=0.018$, CI : [0.012, 0.025]). *Post hoc* comparisons between subtypes within types show some small differences between subtypes comparisons shifting above and below our effect size thresholds within types, however the ordering observed globally (b≥a>c≥d) is preserved in both T4 and T5. Note however that the horizontal (a, b) and vertical (c, d) subtypes are more similar than in PC1.

Within PC3 we find a slightly different result. Here, we observe a medium effect of type ($\eta_{p}^{2}=0.072$, CI : [0.060, 0.086]). *post hoc* comparisons show that T4 ¡ T5 with a medium effect size (Cohen′s_*d*_ = −0.801, CI : [−0.752, −0.849]). This difference may be a result of the two different depths of the respective neuropil layers being innervated by T4 and T5. We find a small effect of subtype $\left(\eta_{p}^{2}=0.032\right)$, CI : [0.023, 0.043]. We additionally find a small main effect of the Type:Subtype interaction term ($\eta_{p}^{2}=0.015$, CI : [0.011, 0.023]). Similarly to PC2, the pattern again emerges whereby b≥a≥c>d.

Given the orderings observed in PC1 and PC2, we fit an ANCOVA model for PC2 with PC1 as a covariate (Fig. 2,e). Here we find a large main effect indicating PC1 as a strong predictor of PC2 ($\eta_{p}^{2}=0.539$, CI : [0.476, 0.600]), with a slope of *β* = −0.848. The Type:PC1 interaction term has a small effect size ($\eta_{p}^{2}=0.021$, CI : [0.014, 0.030]), indicating a difference in slopes between T4 (*β* = −0.835) and T5 (*β* = −0.683). This tells of a stronger negative relationship between PC1 and PC2 in T4 than in T5 (Δβ=−0.151, CI : [−0.135, −0.166], Fig. 2,e).

Together, these results tell us that all T4 and T5 dendrites elongate primarily along the DV axis in both the Medulla and Lobula. However, the extent of this elongation is subtype-specific, although in the same manner between T4 and T5. Interestingly, the observed negative correlation between the dendritic spread along the DV and AP axis indicates that the greater the extent of outgrowth along the DV axis, the lesser the extent along the AP axis in a proportional manner.

#### Dendrite Volume

Having established the shape of T4 and T5 dendrites we next turn to investigate the spanned volume of each dendrite. Previous work has shown that T4 and T5 dendrites span across approximately seven visual columns in both the Medulla and Lobula [10, 11]. This is done by fitting a convex hull to the each individual dendrite morphology (Fig. 2,f). Here we find a small main effect of type ($\eta_{p}^{2}=0.053$, CI : [0.044, 0.064]). *Post hoc* comparisons indicate a medium sized effect indicating that mean convex hull volume is greater in t4 than in T5 (Cohen′s_*d*_ = 0.728, CI : [0.880, 0.773], Fig. 2,f). The effect size for both subtype and type:subtype interaction is not meaningfully large $\left(\eta_{p}^{2}\leq 0.01\right)$.

However, the Medulla is notably larger than the Lobula. In order to account for this difference, we scale dendrite convex hull volumes by the volume of a surface mesh for Medulla layer 10 for T4 and Lobula layer 1 for T5 (see Materials and Methods, Surface Fitting, Volume, and Depth Measurement). Once doing so, all type, subtype, and interaction effect are no longer meaningfully large $\eta_{p}^{2}\leq 0.01$ (Fig. 2,g). This indicates that the mean proportional volume is the same across all types and subtypes, as would be expected from each dendrite spanning seven adjacent columns in space.

We note however that the distribution of T4 volumes is notably more heavy tailed than that of T5 (Fig. 2,f and g; Fig. S1). When we look at the volume of T4 and T5 dendrites plotted spatially, we note a clear structure in T4 not present in T5 (Fig. 2,h; T4: left, T5: right). The tail of larger T4 dendrites clearly cluster in the Ventral-Anterior region of Medulla layer 10, a spatial organisation which is not clearly visible in T5 dendrites within Lobula layer 1. This observed pattern is mirrored in other size-based morphometrics used, including total dendrite cable length, and the number of sections within each dendrite (the number of edges in the underlying tree-graph, Fig. S1). It is interesting to see that each of these size-based metrics is highly correlated, illustrating the consistent relationship between size based metrics show in these dendrites

We have shown here that both T4 and T5 dendrites occupy the same mean proportional volume within their differing neuropils. For T4 dendrites, however, we see a heavy tailed distribution of dendrite size not present in T5, and a spatial organisation favouring larger regions spanned by the dendritic arbour in the Ventral-Anterior region of Medulla layer 10 which is not apparent in Lobula layer 1. The highly correlated relationships observed between convex hull volumes, total cable length, and total dendrite sections, are indicative of coordinated proportional growth. As dendrites expand the volume of their spatial occupancy, they increase their cable length and branching complexity proportionally.

#### Dendrite Depth

**Fig 3.**
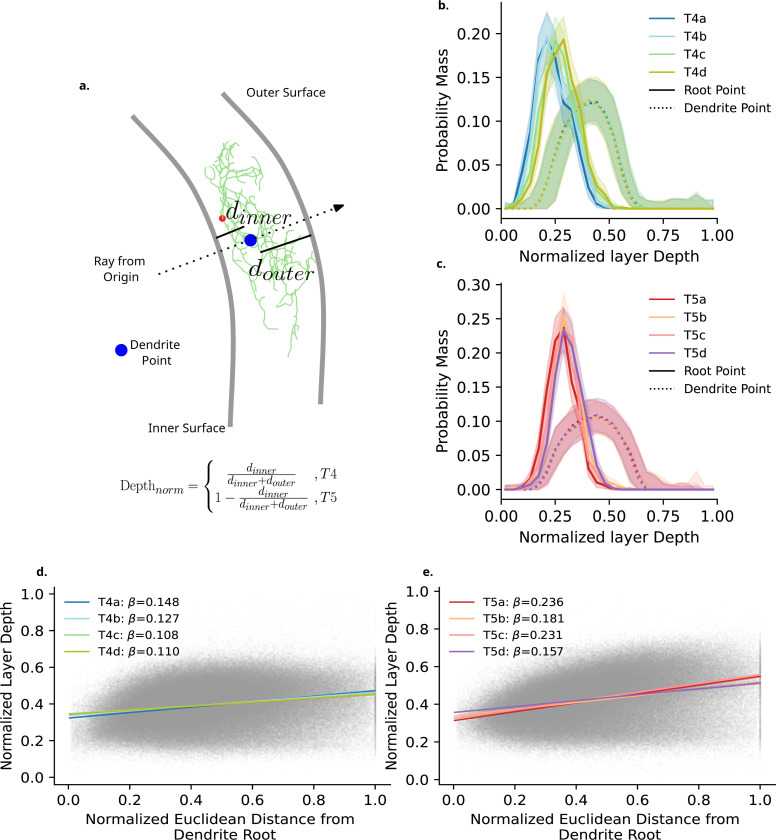
Dendrite Neuropil Layer Specific Depth. a) Schematic illustrating Neuropil specific depth calculation within T4 and T5. Layer surface meshes are generated using all dendrite points, and inner and outer faces estimated using the dot product of a ray from the coordinate origin and the normal of each surface face (See Materials and Methods, Surface Fitting and Volume). For both Medulla Layer 10 and Lobula layer 1, inner and outer surfaces are assigned in the direction of concave curvature. As T5 dendrites innervate from the side of the the outer surface, and T4 from the inner, T5 normalized depths are inverted. b) Distribution of T4 dendrite point depths. Solid lines indicate dendrite roots, with a bootstrapped confidence interval around the probability mass function. Dashed lines represent the distribution of non-root points within dendrites, shaded region show asymmetric MAD within the probability mass function across individuals. c) As in b, for T5 dendrites. d) Multiple Regression showing subtype specific slopes within T4 dendrites illustrating the relation ship between normalized euclidean distance from root (normalised by the maximum per dendrite) and normalized point depth within Medulla layer 10. Root and furthest points within each dendrite (x=0 and x=1) are excluded. e) As d, but for T5 dendrites showing normalized layer depth in Lobula layer 1.

We have focused thus far on the manner by which T4 and T5 dendrites fill space along the DV and AP axes. We turn next to understanding how individual dendrites occupy space along the remaining depth axis. In order to do so, we calculate the normalized neuropil layer depth of dendrite nodes (Fig. 3,a; Materials and Methods, Surface Fitting, Volume, and Depth Measurement).

We first investigate the depth of the first branching point of individual dendrites (Dendrite root, Fig. 3,b and c; solid lines). We find a small main effect of Type ($\eta_{p}^{2}=0.021$, CI : [0.014, 0.029]). *Post hoc* comparisons reveal that T4 dendrites begin branching closer to the surface of Medulla layer 10 than T5 dendrites within Lobula layer 1 with a small to medium effect size (Cohen′s_*d*_ = −0.563, CI : [−0.563, −0.460]). We additionally find a small main effect of Subtype ($\eta_{p}^{2}=0.025$, CI : [0.018, 0.034]). *Post hoc* comparisons reveal that a ≤ b ≤ c ≤ d. This indicates a subtype-specific spatial ordering to the depth at which the dendrites start their branching process. We find no meaningfully large effect of the Type:Subtype interaction. Qualitatively however, we note that our data seems to indicate that T4 a-b subtype dendrite roots are closer to the Medulla layer 10 surface in comparison to c-d (Fig. 3,b) in a more notable manner than is observed in T5 (Fig. 3,c).

When considering the depth of non-root nodes we find no differences between type, subtype, or interaction $\left(\eta_{p}^{2}\leq 0.01\right)$. This indicates that individual dendrites, excluding the start of dendritic branching, occupy the same depth within the neuropil layer regardless of type and subtype, or direction of innervation within the neuropil layer itself.

We next consider the manner by which T4 and T5 dendrites project across their respective neuropil layer. As both Medulla layer 10 and Lobula layer 1 are well approximated by a curved surface, two possibilities exist. Either, dendrites could follow this curvature as they fill space, or they could project across this curvature. In the latter case, we would expect each dendrite to exist primarily on a 2D plane, and fill space at an angle across the neuropil layer curvature. Given the proximity of the dendrite root position to their respective neuropil layer surface we would expect dendrite points further from this root to be closer to the opposing layer surface. By including the Euclidean distance from the dendrite root point, normalised by the maximum within each dendrite, as a covariate predictor for normalised depth, we find a small main effect for this covariate ($\eta_{p}^{2}=0.010$, CI : [0.010, 0.010]) with a slope of *β* = 0.146 (Fig. 3, d and e). With no meaningful effects of type or subtype, this tells us that all T4 and T5 dendrites project across the neuropil layer at a slight angle (Fig. 3,d and e), with increasing depth in the neuropil layer away from the dendrite root. We note however that this particular effect size is at the borderline of our cut-off.

Taken together our analysis shows that the depth at which dendrite subtypes begin arbourising may differ by subtype, more noticeably for T4 than T5 subtypes. The depth of the dendritic arbour itself, excluding the dendritic root, does not differ between types and subtypes. Finally, we find that these dendrites project across their respective neuropils, rather than growing along the curved space.

#### Dendrite Initial Positions

**Fig 4.**
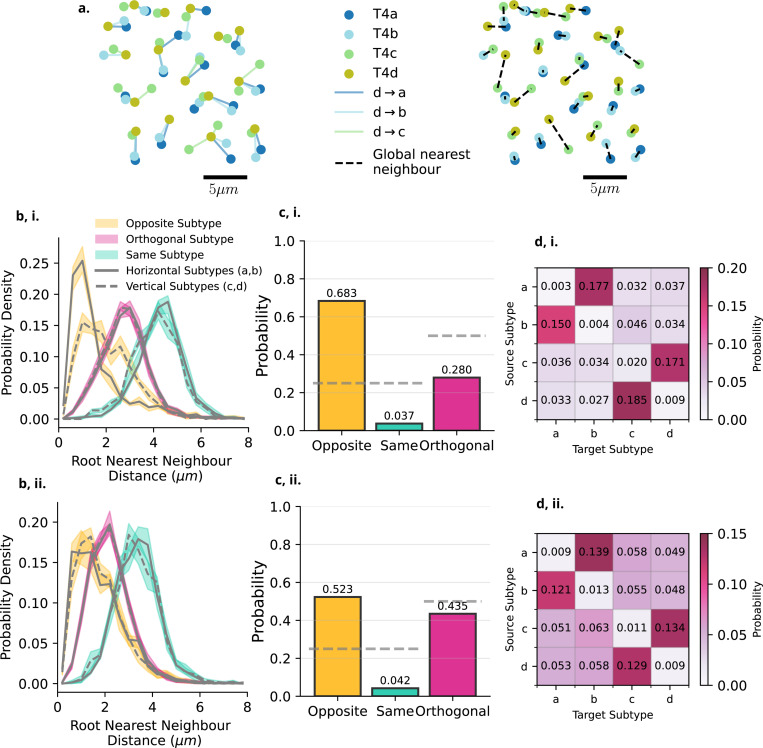
Dendrite root nearest neighbour relationships. a) Illustration of nearest neighbour calculations from a 20*μm* section of dendrite root positions within the central medulla. (*left*) shows subtype specific nearest neighbours from T4d dendrites to T4 a,b, and c subtypes (T4d →T4d are not shown for simplicity. (*right*) Global subtype agnostic nearest neighbour calculations determined by maximum cardinality matching in order to find the unique set of nearest neighbour pairs which minimises the global distance between pairs of dendrite root points. b) Nearest neighbour matching as in a (left). i: Within subtype nearest neighbour distance distributions for T4 dendrites. Dendrite subtypes are grouped into horizontal (a, b subtypes, solid lines) and vertical (c, d subtypes, dashed lines). ”Opposite” distributions denote opposingly oriented subtypes (eg, a → b). ”Same” subtypes denote same subtype combinations (eg, a → a). ”Orthogonal” denotes orthogonally oriented subtypes (eg, a → c and a → d). ii: As in bi, but for T5 dendrites. c) Nearest neighbour matching as in a (right). i: Subtype agnostic probability of global nearest neighbour being of the ”Opposite”, ”Same”, or ”Orthogonal” group within T4 dendrites. Dashed lines denote approximate chance probability for each group (0.25 for ”Opposite” and ”Same” groups, 0.5 for ”Orthogonal” groups). ii: As in c i, but for T5 dendrites. d) Nearest neighbour matching as in a (right). i: Subtype specific nearest neighbour subtype probabilities within T4 dendrites derived from global population nearest neighbour calculations. ii: As in di, but for T5 dendrites.

Each column within the Medulla and Lobula contains four T4, or T5 dendrites, one of each subtype. With the absence of neuropil specific spatial column information, we investigate the pairwise nearest-neighbour relationships between dendrite root nodes. This informs us about any possible spatial structure in the initial starting positions of dendritic arbourisation. We find both the subtype-specific nearest-neighbour pairings for dendrite root nodes, and additionally the global subtype-agnostic unique pair assignment which minimises the total distance between pairs of dendrite root nodes (Fig. 4,a; Materials and Methods, Dendrite Root Nearest Neighbour Matching). Alongside subtype labels, we additionally group data into ”same” (e.g a→a), ”opposite” (e.g a→b), or ”orthogonal” (e.g a→c and a→d) groups.

When looking at the subtype specific distribution of nearest-neighbours, we find that in all cases the ”opposite” subtype is the closest nearest neighbour for any given dendrite’s root, and the ”same” subtype is always the furthest, with ”orthogonal” subtypes occupying the middle distance, with no separation between which of the ”orthogonal” subtypes is closer (Fig. 4,b). Additionally, we observe that within T4 but not T5 dendrites, the horizontal (a,b; Fig. 4,b(i), solid lines) subtypes and vertical (c,d; Fig. 4,b(i) dashed lines) distributions differ. The vertical subtypes show a greater distance between their nearest neighbours in relation to the horizontal, which is not the case in T5 dendrites (Fig. 4,b (ii)).

Based on the subtype agnostic nearest-neighbour pairing, we can additionally calculate the probability of a given nearest neighbour being of the ”Opposite”, ”Same” or ”Orthogonal” classification (Fig 4,c). We find that in both T4 and T5 the nearest-neighbour root node is most likely to be of the ”Opposite” classification in a manner which is well above chance. This pattern also extends to subtype-specific pairings (Fig. 4,d). Here, for both T4 and T5, we see that the most probable nearest neighbour is of the opposite subtype (a to b, c to d), and the same subtype the least likely (Fig. 4,d diagonal), with the orthogonally oriented subtypes positioned between the opposite and same subtypes.

This indicates a clear spatial organisation in the dendrite root node positions, highlighting the potential importance of the initial pattern of innervation before dendritic arbourisation. How this result relates to the spatial position of dendrite root nodes within optic lobe columns remains to be determined however.

### Dendrite Orientation and Geometry

The asymmetric orientation of T4 and T5 dendrites has been well established [10–12, 14, 35]. Beyond this orientation however, the high resolution of these data allows for the analysis of the orientation of individual dendritic sections, and the geometry of bifurcations. We have already observed that all dendrites, regardless of subtype, occupy an elliptical space oriented along the DV axis. This alone does not show their characteristic directionality however. The manner by which individual sections within each dendrite are oriented tells us how this asymmetry may emerge, and if outgrowth during development is coordinated along the DV/AP axes, or independently.

#### Dendrite Orientation

**Fig 5.**
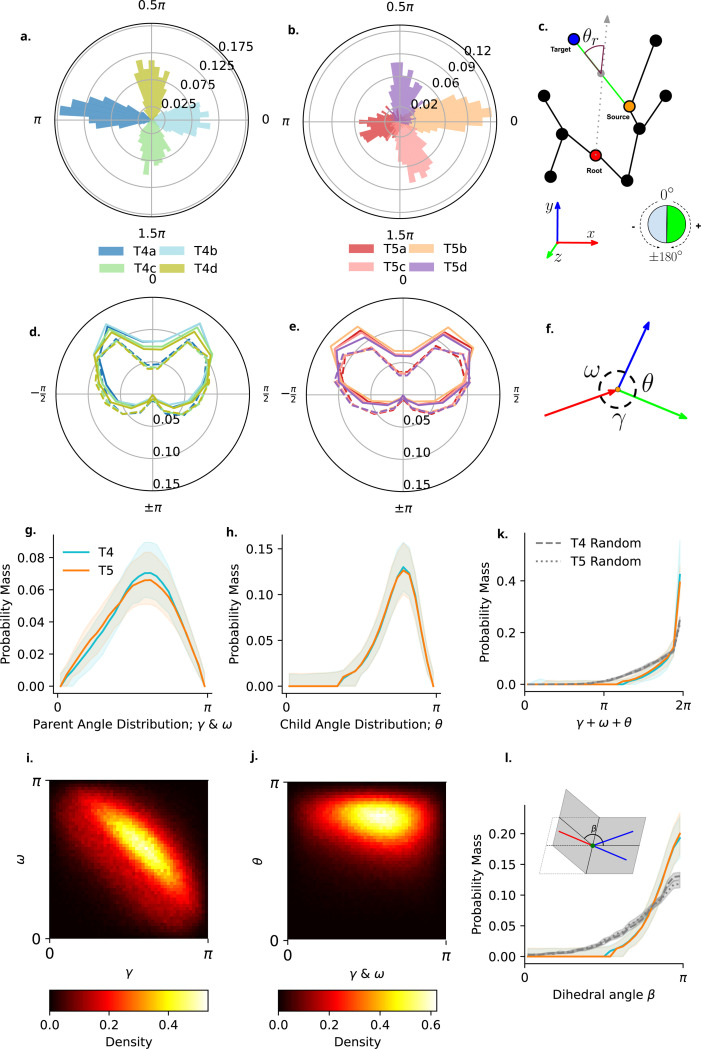
Dendrite orientation and Geometry. a) Distribution of mean section angles for each dendrites relative to the Anterior-Posterior axis, coloured by subtype. b) As in a, for T5 dendrites. c) Schematic illustration of radial angle calculation. Note that $\theta_{r}=0$ denotes a section perfectly aligned with the ray cast from the dendrite root (gray dashed arrow). As such, segments where the source node is the dendrite root have $\theta_{r}=0$ and are excluded from analysis. Plane normal orientations used for calculation of the sign of $\theta_{r}$ are oriented in the same direction to ensure accurate signs for $\theta_{r}$. d) Radar plot showing distribution of section radial angles within T4 subtypes. Solid lines show internal dendrite sections, and dashed lines denote external leaf sections. e) As in d, but for T5 dendrites. f) Schematic illustration indicating notation of bifurcation angular components. The red arrow denotes the parent section, and blue and green indicate the two child sections. γ and ω show the angles from the parent section to either of the two child sections. θ denotes the angle between the two child sections. g) Distribution of parent to child angles γ and ω within T4 and T5 dendrites. Shaded region show asymmetric MAD within the probability mass function across individuals. h) Distribution of child angle θ for T4 and T5 dendrites. Line colours are the same as in g. i) Joint probability distribution illustrating inverse relationship between parent to child angles γ and ω. Data for type and subtype are collapsed together. j) Joint probability distribution showing relationship between Parent to child angles γ and ω with child angle θ. k) Distribution of the sum of bifurcation angular components (γ, ω and θ) for T4 and T5 Dendrite bifurcations. Gray dashed (T4) and dotted (T5) lines show bifurcation angular component sums for randomly generated bifurcations for comparison (See Materials and Methods, Geometry Analysis). l) Distributions of Dihedral angle β, used as a measure of 3D bifurcation flatness for T4 and T5 dendrites. Gray dotted and dashed lines facilitate comparison against randomly generated bifurcations as in k.

For each dendrite we can calculate the mean angular orientation of individual sections within the dendrite, which shows a clear organisation into the four known subtypes in both T4 and T5 (Fig. 5, a and b). This dendrite-section-based analysis of orientation is in line with similar analyses conducted on the orientation of differing Strahler order sections [14]. Interestingly here the distribution of T5a dendrite orientations is, although still oriented, far less clear than all other subtypes (Fig. 5,b). This may relate to the previously noted more centralised dendrite root positions (Fig. 2,a).

We next analyse the radial orientation sections within the dendrite. In order to do so we calculate the signed radial angle of each dendrite section [36] (Fig. 5,c; Materials and Methods Geometry Analysis). Given the dominant elongation along the DV axis observed, coupled with four orientations of subtypes, we wish to know if either, dendrite sections align with the DV and AP axis, or alternatively, if sections orient radially outwards from the dendrite root node.

We find a tightly symmetrical distribution of radial section angles with no difference between subtypes (Fig. 5, d (T4) and e (T5)) oriented around $∼\pm \frac{\pi }{4}$. We have classified individual sections as ”internal” or ”external” within this analysis (See Materials and Methods, Neurons as Tree Graphs). This denotes whether a section is contained within the dendrite, starting at the dendrite root or at a branch point, and ending at a branch point (internal), or if a section terminates at its end point (external). We note that T5 dendrites show a broader set of radial orientations than T4, likely caused by growth in differing neuropils and different local spatial constraints. We observe that radial orientation of internal and external sections differs, with internal sections (Fig. 5, d,e; solid lines) showing tighter radial angles closer to 0°, more closely aligned with the vector from the dendrite root in comparison to external sections (Fig. 5, d,e; dashed lines).

Although previous work has identified a radial angle distribution centred around 0° [36], we find that within T4 and T5 dendrites, sections are still oriented consistently outward from the start of branching, however distributed symmetrically around 0° at $∼\pm \frac{\pi }{4}$. This shows that individual dendrite sections, rather than being oriented along or across the DV or AP axis, are oriented outwards from the initial branching point in a similar manner for both T4 and T5 dendrites as well as their subtypes. We also note that, although still oriented outwards, external sections show a broader distribution and are less constrained by the radial orientation away from the definite root node in comparison to internal sections (Fig. 5, d and e; solid lines compared to dashed). This indicates that the manner in which these dendrites orient individual sections, and by extension their asymmetric outgrowth, is to project outwards from the start of dendrite branching in a systematic and conserved manner across subtypes, as opposed to orienting along the DV or AP axis.

#### Dendrite Bifurcation Geometry

As our next step in building our understanding of the branching structure of T4 and T5 dendrites, we analyse the geometric structure of bifurcations in the dendrite arbourisation. Note that we limit our analysis strictly to bifurcations, although some trifurcations, or higher are present within the dataset (∼ 6 trifurications per dendrite). Additionally, rather than calculate angles based on the whole section between root, branch, and leaf nodes, we limit analysis to 0.1*μm* region around the branch node, using the full skeleton reconstruction as opposed to the reduced tree-graph representations (See Materials and Methods, Neurons as Tree Graphs; Geometry Analysis). Each bifurcation is split into three angular components γ and ω, the angles between the parent branch and each of its two children, and θ the angle between the two child branches (Fig. 5,f). As γ and ω are ordered arbitrarily they are collapsed together for analysis unless otherwise noted.

When first comparing the distributions of these angular components, we find no meaningfully large effects of type, subtype, or their interactions ($\eta_{p}^{2}\leq 0.01$, Fig. 5, g and h). We next compare the pairwise relationships between angular components. Here we find that γ is a predictor of ω with a small effect size ($\eta_{p}^{2}=0.056$, CI : [0.054, 0.058]), and a negative slope (*β* = −0.553, Fig. 5,i). No other effects or interactions here have an effect size greater than our minimum cutoff $\left(\eta_{p}^{2}<0.01\right)$. No such relationship is found when comparing either γ or ω with the child angle θ (Fig. 5,j; all $\eta_{p}^{2}\leq 0.01$).

We next investigate the planarity of dendritic bifurcations, a property which has been observed in a number of vertebrate neurons [37–39]. Additionally, as the occupied space of T4 and T5 dendrites is naturally flat, we create a simple null model by drawing sets of three unit vectors with a parent vector oriented along the negative x-axis and the two child vectors oriented along the positive x-axis. We additionally scale the distribution of points to align with the average dispersion along each axis observed in our data for T4 and T5 separately, derived from the PC weighting (see Materials and Methods, Geometry Analysis).

First, we look at the sum of angular components for each bifurcation. The sum of angles at a bifurcation in 3D is restricted in three ways: (i) each angle will be smaller than or equal to 180°, (ii) the largest angle will be ≤ the sum of the other two, and (iii), the sum will be ≤ 360° [37]. The sum of angular components within bifurcations peaks at 2π, with no meaningfully large differences between types, subtypes or interaction $\left(\eta_{p}^{2}\leq 0.01\right)$. The observed distribution is more heavily dominated by values close to 2π than observed in our random null model (Fig. 5,k).

Second, we compute the Dihedral angle β for each bifurcation, recommended by [37] as a measure of bifurcation flatness (Fig. 5,l insert; Materials and Methods, Geometry Analysis). Again, no meaningfully large difference is found between type, subtype, or interaction $\left(\eta_{p}^{2}\leq 0.01\right)$. Additionally, we note that the observed distribution is more concentrated around π in comparison to our simple random null model.

Taken together, these results indicate that bifurcations within T4 and T5 are identical across both types and subtypes, with individual bifurcations being flatter than expected by chance and occupying a circular space. The inverse relationship observed between γ and ω is indicative of an optimal spatial embedding whereby both child sections are oriented forward from the parent section and maximally distant from eachother. Due to the summation of angles to 2π and bifurcation planarity, the child angle θ then occupies the remainder of the circular space.

### Dendrite Topology

Our final section of analysis considers the topological and graph-theoretical structure of T4 and T5 dendrites. Analysis at this level, which is concerned with the underlying structure of dendrites, is less influenced by external factors, such as mechanistic forces dictated by their external environment. As such, we can infer from these metrics details of the internal developmental process for each dendrite, independent of external signals. Conservation of structure at this level is seen as further evidence in favour of a shared developmental mechanism across types and subtypes, independent of variability introduced between neuropils.

#### Dendrite Graph Structure

**Fig 6.**
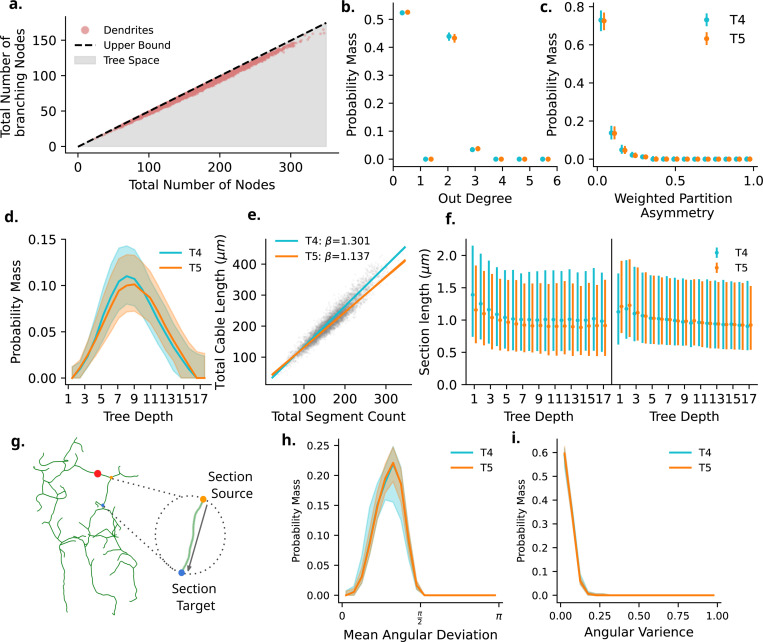
Graphical Structure of Dendrites. a) Scatter plot showing the relationship between total number of nodes and number of branching (internal) nodes within a dendrite tree graph. Gray filled in space denotes the region of possible tree-graphs, where by outside this region a given graph will no longer be a tree-graph. Black dashed line denotes a binary tree. b) Distribution of node out-degree for T4 and T5 dendrites. Nodes with out-degree of 0 are leaf nodes, and and out-degree of 2 show bifurcations. No nodes with an out-degree of 1 exist in the reduced tree-graph representations used (See Materials and Methods, Neurons as Tree Graphs). Error bars show asymmetric MAD of probability mass functions across individual dendrites. c) Distributions of weighted partition asymmetry across T4 and T5 dendrites (See Materials and Methods, Branching Structure and Symmetry Quantification). Values of 0 show symmetric branching and 1 show asymmetry. Error bars show asymmetric MAD of probability mass functions across individual dendrites. d) Distribution of external (leaf node) counts at increasing dendrite tree-graph depths. Error bars show asymmetric MAD of probability mass functions across individual dendrites. e) Relationship between number of dendrite sections and total dendrite cable length in T4 and T5 dendrites. f) Section length with increasing tree-graph depth for both T4 and T5 dendrites in internal (left) and external (right) sections. Error bars show asymmetric MAD of probability mass functions across individual dendrites. g) Schematic illustration showing curvature of individual segments around the euclidean vector between source and target nodes of the section. ”Raw” dendrite skeletons are sampled at 10*nm*, so a single dendrite tree-graph section is comprised of multiple edges, and the euclidean distance between the source and target of dendrite sections is less than the sum of edge weights. h) Distribution of mean angle between individual edges and their respective euclidean section vector. Error bars show asymmetric MAD of probability mass functions across individual dendrites. i) Angular variance of raw dendrite edges around each dendrite section euclidean vector. Error bars show asymmetric MAD of probability mass functions across individual dendrites.

We first determine the nature of the underlying tree-graph for T4 and T5 dendrites. At one extreme, a star graph contains a single internal node and all other nodes are external, leaf nodes. For such a graph the number of branching nodes (I) is equal to 1. At the other extreme, a full binary tree is a tree-graph whereby all internal nodes have an out-degree of two. Within the reduced tree-graphs used here where all nodes with a single parent edge and a single child edge are removed, a full binary tree will have $I=\frac{n-1}{2}$ for n total nodes (see Materials and Methods, Neurons as Tree Graphs). When plotting the relationship between total number of dendrite nodes and total number of branching nodes (Fig. 6,a) we find that all T4 and T5 dendrites are slightly beneath this upper bound for a full binary tree. Statistical analysis identifies the total number of nodes as a predictor of total number of branch nodes with a large effect size ($\eta_{p}^{2}=0.972$, CI : [0.967, 0.978]) with a slope of *β* = 0.479. We find no meaningfully large differences between other main effects or interactions $\left(\eta_{p}^{2}<0.01\right)$.

When observing the out-degree distribution of dendrite tree-graphs we see that these dendrites are dominated by nodes with an out-degree of 0 (all leaf nodes) and 2 (bifurcating branch nodes). Interestingly, there is a highly consistent but small number of trifurcations within the dataset (∼ 6 per dendrite). We note that these trifurcations could be a result of the skeletonization process, although their consistency leads us to believe otherwise. We find no meaningfully large differences between types, subtypes or type:subtype interactions $\left(\eta_{p}^{2}\leq 0.01\right)$.

We next consider the symmetry of dendrite tree-graphs. Tree asymmetry has long been used as a metric in the analysis of neuron morphology [40–42]. Symmetry here refers to the symmetry of branching through the dendritic tree and is calculated at each branch node. As an example, if one pictures a bifurcation whereby the left downstream node is a termination, and the right contains the entirety of the downstream subtree which follows this same right-dominated rule, this bifurcation would be asymmetric and as such have a tree asymmetry value of 1. On the other hand, if all branching nodes downstream from a bifurcation are also bifurcations, this subtree would be symmetric, and as such have a tree asymmetry value of 0. We implement here a weighted tree asymmetry metric, similar to [42] (See Materials and Methods, Branching Structure and Symmetry Quantification). Rather than using a single summary statistic per dendrite, we show the full distribution of scores across a single dendrite, showing the clear symmetric nature of T4 and T5 dendrites (values close to 0 are closer to symmetric, Fig. 6,c). This is in line with previous observations [40, 42, 43] of tree symmetry in neuron morphologies. Again, no meaningfully large differences are found for type, subtype, or type:subtype interactions $\left(\eta_{p}^{2}\leq 0.01\right)$.

We note that external leaf nodes are distributed throughout tree graph depths (Fig. 6,d), again with no differences observed between type and subtypes $\left(\eta_{p}^{2}\leq 0.01\right)$. Much like the relationship between total dendrite cable length and dendrite convex hull volume (Fig. S1, c), there is a strong relationship between the total dendritic cable length and the number of individual segments within each dendrite (Fig. 6,e). With the number of segments as a covariate of total cable length we find segment count is a strong predictor of total cable length ($\eta_{p}^{2}=0.628$, CI : [0.584, 0.675]). We additionally find a small effect size for the Type:Section count interaction ($\eta_{p}^{2}=0.019$, CI : [0.013, 0.026]). Together, this tells us that we observe a small difference in slopes between T4 and T5, indicating each dendrite section corresponds to 1.300*μm* in T4 and 1.136*μm* within T5. We find no other differences between subtypes or interactions.

Together, these results speak of a highly conserved topological structure for all T4 and T5 dendrites, as well as their subtypes. We did not find any distinguishable characteristic within this structure which would be in favour of arguing for differences between subtypes mediated by differing orientations, or neuropil specific differences between T4 and T5 with the exception of the slight differences in slopes observed in the relationship between section counts and total cable lengths, likely mediated by the neuropil.

#### Dendrite Section Lengths

We next turn to investigate any differences in the individual sections between T4 and T5 and their subtypes. As was done previously when considering the radial orientation of individual sections, we again include the internal or external nature of individual sections within our analysis. We first investigate the decay of section lengths as a function of tree depth (Fig. 6,f). Tree depth here refers to the number of (unweighted) steps taken from the root of the dendritic tree. We find no meaningfully large differences between type, subtype, source, or interactions (all $\eta_{p}^{2}\leq 0.01$). We note, however, that sections closer to the dendrite root (with lower depth), particularly within internal sections, appear marginally longer before the section lengths stabilise. This observation of an initial decay followed by stabilization has been observed previously in mouse Purkinje cells [44], and in line with an optimal wiring and conduction time trade-off, which would imply proximal longer runs and distal finer segmentation [8]. T4 internal section lengths also appear qualitatively greater than T5 across all tree depths (Fig. 4,f; left).

The individual section within a dendrite are a collection of short edges which curve around the individual root, branch and leaf nodes within the tree graph (Fig. 6, g). We would like to understand how much deviation there is from the shortest vector between points as the dendrite section projects between them. As such, we measure for each section the mean angular deviation between individual tree graph edges and the Euclidean vector between the source and target point of the section, and the angular variance of the individual edges. We find no differences between types and subtypes $\left(\eta_{p}^{2}\leq 0.01\right)$, showing the curvature within individual sections is tightly conserved again across types, subtypes, and sources.

**Fig 7.**
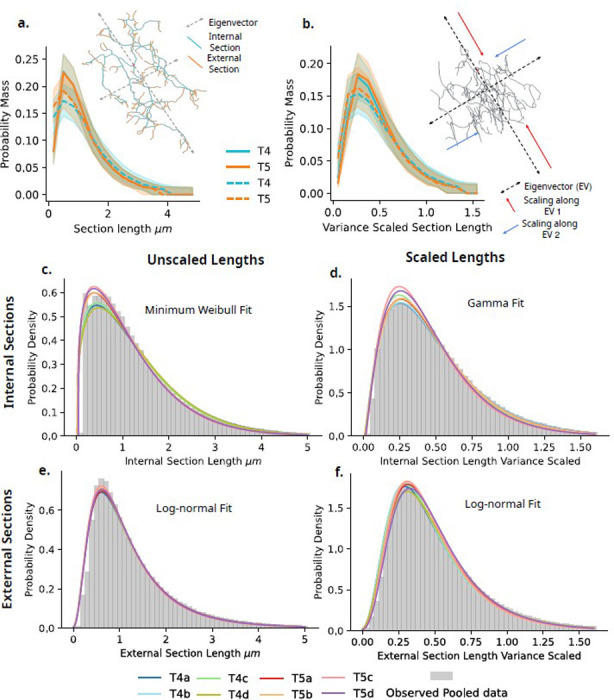
Dendrite Section length Distribution and Explorative Distribution Fitting. a) Distribution of dendrite section lengths for T4 and T5 dendrites, with dashed lines showing internal sections and solid lines showing external leaf sections. Schematic (insert) shows example neuron with internal (cyan) and external (orange) sections coloured for illustration, along with first two eigenvectors. b) Distribution of section length as in a, but for variance scaled dendrites (See Materials and Methods, Alignment and Scaling). Dendrites are individually scaled to have equal variance along each eigenvector in order to account for neuropil specific differences. Schematic (insert) illustrated variance scaling along eigenvectors. c) Best fitting tested distribution for unscaled internal section lengths (minimum Weibull distribution) d) Best fitting tested distribution for scaled internal section lengths (gamma distribution) e) Best fitting tested distribution for unscaled external section lengths (log-normal distribution) f) Best fitting tested distribution for scaled external section lengths (log-normal distribution). Gray histograms show the probability density for all pooled data. Fitted probability density functions are shown for all subtypes colour coded by key at the base of the figure. Error bars in a,b, show asymmetric MAD of probability mass functions across individual dendrites.

We next investigate the distribution of individual section lengths across T4 and T5 and their subtypes. As well as ”raw” section lengths, we additionally scale individual dendrites along their principal axis to have equal variance, creating uniform spherical dendrites without changing the topological structure. The intention of this scaling is to remove the influence of the local dendrite environment, such as external mechanistic forces, or the confines of the neuropil layer from the length of dendrite sections, without altering the topological structure. Ideally, this results in a dendrite representation independent of external influence (Fig. 7,a and b).

Interestingly, in both original units and scaled datasets, we find no meaningful differences between type, subtype, source, or their interactions $\left(\eta_{p}^{2}\leq 0.01\right)$. Due to the high skew observed in these data, we also test log-transformed values, which have the same result $\left(\eta_{p}^{2}\leq 0.01\right)$. Our analysis here again shows a highly conserved structure with no meaningfully large differences between T4 and T5 dendrites and their subtypes.

#### Section Length Distributions

Previously, measures of dendrite and axon section lengths have been observed generally with a range of right-skewed distributions [42, 45–49]. However, understanding the specific nature of the observed right-skewed distribution tells us something of the underlying mechanistic process. For example, models of multiplicative dynamics have been shown to directly give rise to the observed log-normal distribution of spine size in the auditory cortex of mice [49].

In order to better understand the specific distributions observed here, we perform an exploratory analysis to determine which from a set of six distributions best fits the observed section length data. We choose to compare exponential, log-normal, gamma, Wald, log-logistic, and minimum Weibull distributions. Although we note that an exponential distribution is a special case of the gamma distribution with k=1, we keep the two distributions distinct for clarity. Each of these distributions is an example of a right-skewed distribution, and can be related to differing generative processes. We fit all six distributions to each type, subtype, and source combination for both raw and variance scaled section length data. Distributions are compared using the Bayesian Information Criterion (BIC). Interestingly, there is always a single consistent best fitting distribution for types and subtypes.

For raw section lengths within internal sections, which relates to the branching interval, we find that the data is best fit by a minimum Weibull distribution. Such a distribution has been used previously to fit inter-spine interval data within macaque prefrontal cortex [50]. The minimum Weibull distribution is typically used to model time-to-event, and is often characterised by this time-to-event being variable, for example with an initial longer interval before stabilising at a faster rate [51]. Interestingly, this is in line with our previous observation of internal section lengths closer to the dendrite root (Depth ≤ 3) being marginally longer, before stabilising (Fig. 6,f; Right). Additionally, fitted distributions show a clear distinction between T4 and T5 (Fig. 7,c), which is also in line with the small qualitative difference observed in internal section lengths as a function of tree depth (Fig. 6,f). This may relate to the fact that T4 and T5 dendrites initially occupy a concise region spanning approximately a single column up to 36 hours after pupa formation (APF) before their dendritic orientation emerges, spanning across the remaining six columns between 36–72 hours APF [17], indicating a change in the manner of dendrite outgrowth tied to the external spatial environment during development.

For variance scaled internal sections, which are assumed to be independent of external factors, data is consistently best fit by a gamma distribution (Fig. 7,d). The gamma distribution relates to a process involving the necessity for multiple events to occur before a branching event takes place. Giving a more concrete example, this implies that a branching event will occur during growth after n sub-events, which are exponentially distributed, have first occurred [52]. The fitted distribution to each group here shows greater variability in comparison to other fits, although there is no clear structure to this variability (Fig. 7,d). When branching is treated as a Poisson process, and multiple events are required before a stable branch occurs, we would expect to observe a gamma distribution as a result [53]. Empirically fit models which treat branching in this manner have previously been used to accurately capture the observed distribution in real data [54–56]. This assumes a stable dendrite branch requires some number of molecular sub-events before formation. Actin nucleation, specifically, transient, localized Arp2/3 recruitment, precedes branch formation in *Drosophila* DA neurons [57], as an example of one such potential molecular sub-event. Further, microtubules have been shown to transiently invade branch precursors, providing a pathway for additional material required for stabilisation, initiating changes in dendrite spine morphology [58]. MAP2c in cultured hippocampal neurons has also been shown to promote stabilisation of neurites, through the coupling of F-actin and microtubules, consolidating stabilisation [59]. Our data is indicative of a complex multi-event mechanism which governs branching within these dendrites.

Finally, for both raw and scaled external section lengths, we find data is consistently best fit by a log-normal distribution for all types and subtypes, with little variability between fitted distributions (Fig. 7,e and f). A log-normal distribution has been previously related to dendrite spine outgrowth and lengths [49], as well as axonal tree section lengths [42]. The log-normal distribution is a result of a central limit theorem-like process resulting from the product, rather than the sum, of multiple samples [60, 61]. Dendritic spine lengths have previously been observed to follow this type of distribution, both experimentally and within models [3, 49, 62]. How this may relate mechanistically to the outgrowth of dendrite external sections is not as clear however. Live imaging has shown stochastic switching between growth, paused, and shrinking states in an episodic manner within dendrite tips [63]. This dynamic state switching, coupled with the available resources in the dendrite tips, could, in principle, generate the multiplicative effects required to produce the observed log-normal distribution [42].

Together, this exploratory analysis indicates fundamentally differing generative processes underlying branching rates and termination rates. Further, the external environment seems to have an impact on the internal section length, analogous to the inter-branch interval, in a neuropil specific manner, potentially relating to differing rates of spatial occupancy dependent on the local columnar structure during development. That our scaling has no impact on the log-normal nature of external section lengths indicates that the mechanism of outgrowth and termination of arbours is shared across T4 and T5, and independent of external environmental factors.

## Discussion

By leveraging the high resolution morphological data available through new EM reconstructions of all T4 and T5 dendrites we are able to offer a quantitative characterisations of similarities and differences between T4 and T5 dendrites, as well as their subtypes at a level not available through typical imaging techniques.

### Dendrites are Structurally Homogeneous

Given the distinct characteristic orientation of T4 and T5 dendrite subtypes [13], one may assume that this would lead to morphological differences within the dendrite branching structure. We, however, observe consistent homogeneity in all geometric, and graph-theoretical measures across all T4 and T5 dendrites, as well as their subtypes.

The dendrites of T4 and T5 neurons, and their subtypes, are all well characterised by the same close to binary and symmetric tree-graph with little variability across both neuropils and subtypes (Fig. 6,a and c). Interestingly, the deviation from a binary tree manifests in a small but consistent proportion of trifurcations (Fig. 6,b). Their consistency leads us to believe that they are less likely to be a direct result of the skeletonization procedure. This may indicate that they are biologically meaningful, for example, being Steiner points which minimize the total dendritic cable [64], however further analysis is needed in order to determine if this is the case.

We also observe a striking similarity in the geometry of dendritic branching, even though individual subtypes are globally oriented (Fig. 5). The angular components of all dendrite bifurcations are indistinguishable, as is their planarity, which is flatter than would be expected by chance in a similarly confined space. The implication being here that the spatial placement of child sections at a bifurcation is independent of type and subtype, and shared across all dendrites. This planarity is likely a result of additional constraints on branching geometry. Previous work has related the flatness of bifurcations with both the stochastic process of connecting to the closest point in space randomly [65], and being an emergent property of the local 3D elastic tensions in neurites, where distal parts of branches develop force equilibrium when anchored to the substrate [37]. Either mechanism is plausible here. Further, our results indicate a simple rule whereby the two child sections are maximally separated from each other. This is due to the relative planarity of these bifurcations, coupled with the negative correlation between both parent and child branching angles. This maximal partitioning may relate to self-avoidance mechanisms, as well as optimal spatial embedding.

These dendrites also show similar spatial occupancy, shown in their elliptical structure (Fig. 2,a) and consistent elongation along the Dorsal-Ventral axis regardless of subtype (Fig. 2,c), as well as neuropil layer depth innervation (Fig. 3). Once appropriate scaling is applied, the mean volume of these dendrites additionally does not differ (Fig. 2,g), in line with the importance of the spatial occupancy of ∼ 7 columns and necessities of motion detection to integrate across multiple adjacent visual points [10, 11].

Together, we conclude that T4 and T5 dendrites, and their subtypes, are structurally indistinguishable from each other.

### Target Neuropil Impacts Spatial Occupancy and Initial Conditions

Although the structure of individual dendrites is highly conserved across types and subtypes, there remain some insightful differences. Of particular interest is the differing distribution shape and spatial organisation in all size based metrics. Specifically, the population of T4 dendrites shows a heavy tail not present in the comparably symmetric T5 distribution (Fig. 2,f; Fig. S1,a and d). The tail of larger T4 dendrites is focused within the Ventral-Anterior region of Medulla layer 10 (Fig. 2,h; Fig. S1, b and e). It has been previously observed that within the compound eye, across *Drosophilidae* species, larger lenses are present within the Ventral - Anterior region [22–24]. The observed spatial distribution of T4 dendrites could be indicative of a similar pattern being conserved in Medulla columns but not within Lobula columns. In order to investigate this hypothesis however, additional analysis is needed which includes the spatial density of synapses, and neuropil specific spatial column annotations.

We also observe differences between horizontal and vertical subtypes in terms of the dendritic root position. Our analysis of dendrite root depths implies that horizontal T4 subtypes may start branching closer to the layer boundary in comparison to T4 horizontal subtypes, a trend which is not clear in T5 dendrites (Fig. 3, b and c). In addition to this, we see a clear difference between nearest-neighbour partner in T4 horizontal and vertical subtypes when considering oppositely oriented nearest-neighbours, whereby horizontal subtypes are closer nearest-neighbours than vertical (Fig. 4,b i). This trend is not present in T5 neurons (Fig. 4,b ii).

Together, these results show subtle differences which highlight first, possible structural differences between the Medulla and Lobula, with the Medulla potentially mirroring the eye structure in a way which the Lobula does not. Second, the positions of initial dendritic branching, as well as the pairwise relationships, specifically when considering the opposing subtype nearest-neighbour, differ along the horizontal and vertical axes within T4 and the Medulla, but not T5 within the Lobula.

### The Emergence of Orientation

Given the homogeneity of dendrite structure across subtypes and types, it is important to pinpoint exactly what is meant by their characteristic orientation. At some stage during development, symmetry breaking must occur in order to ensure that an individual dendrite spans the region of visual space required for motion detection.

Given the shared elliptical-like spatial occupancy of all subtypes, with dominant elongation along the Dorsal - Ventral axis, the important feature determining orientation within the spatial embedding of dendrites appears to be the centrally offset position of the start of dendritic branching within its ultimately occupied elliptical region (Fig. 2,a). When filling out this elliptical space, the local geometry and orientation of sections away from the initial branching position is conserved across types and subtypes, however the mean orientation of dendrite sections within the global space is clearly subtype specific and directed (Fig. 5).

To illustrate this process, consider an ellipse in which a point is randomly placed at an off-centre location. If short lines a drawn within the ellipse, oriented radially away from the point but directed towards the boundary, the average angle of these lines relative to a global reference will be oriented. While T5a neurons may differ in terms of the position of their dendritic root within the spanned elliptical region, it is evident that the initial branching positions for all other subtypes exhibit a consistent offset relative to the occupied space. Specifically, dendrite roots in the horizontal subtype are positioned centrally within the lower and upper regions of their spanned area, whereas vertical subtype roots are located within the central left or right regions (Fig. 2,a). This configuration enables dendritic orientation within the occupied space, while preserving the structural homogeneity independent of overall orientation. The developmental mechanisms that govern this process in these dendrites remain to be determined however.

### Insights into Dendrite Development

First of all, given the consistent homogeneity of dendrite structure, we reason that the developmental algorithm across T4 and T5 is likely shared. Our analysis indicates a developmental process whereby new dendrite arbours orient outwards from the initial branching point to occupy space in an optimal manner at equivalent rates (Fig. 6) and with shared geometry within both T4 and T5, and their subtypes (Fig. 5). This, alone, would produce a circular dendrite, and it remains unclear how the subtype-specific orientation emerges.

We also observe a subtype, but not type, specific relationship in the extent of dendrite innervation along the dorsal-ventral (DV) and anterior-posterior (AP) axes. Vertical (c,d) subtypes show a greater elongation along the DV axis, and narrower width along the AP axis, a trend which is smaller within the horizontal (a,b) subtypes (Fig. 2,d). Interestingly, however, the difference between elongation along the DV and AP axis is negatively correlated (Fig. 2,e), indicating that as each dendrite extends along the DV axis, it extends less along the AP axis proportionally. This could be evidence of a cost-constrained mechanism limiting the total size of dendrites, coupled with dominant, subtype-specific, elongation along the DV axis.

A likely contender for the emergence of orientation during development is that subtypes make use of chemical gradients. The identification of chemical gradients within the Medulla and Lobula during development which may be related to T4 and T5 development have remained elusive however. One contender would be the identified DWnt4 and DWnt10 A/P gradient identified within the Medulla during development [66]. However this gradient is observed at 25 hours after pupa formation (APF), and not present at 45hrs APF. This is an issue, as the dendritic orientation of T4 and T5 dendrite subtypes does not start to emerge until later than 36hrs APF [17].

Given the absence of a definitive chemical gradient, we propose two potential local cues which could influence dendritic orientation in light of our analysis which would allow for maintained structural conservation. Our analysis shows that dendrites innervate their target neuropil in a subtype specific manner, with opposingly oriented dendrites positioned as nearest neighbours (Fig. 4). Previous work suggests that these nearest neighbour partners likely originate from the same neuroblast (NB) [16]. Additionally neurons from the same NB are able to distinguish themselves from other neurons from the same lineage [67, 68]. During the initial dendritic outgrowth phase where dendrites tend to be confined within a single column [17] dendrites originating from the same NB may provide attraction or avoidance cues determining later orientation and outgrowth. Second, each dendrite densely overlaps in space. During development, neurons will form synapses promiscuously and, taken out of their developmental context, form any synapses they can [69]. This implies that dendrites will compete spatially for synapses. For two dendrites which initiate branching close together, this competition will be reduced in the opposing direction, facilitating directed outgrowth.

While such promiscuity could disrupt the precise synaptic compartmentalisation observed within these dendrites [10], we have shown that they orient across, not along, the neuropil depth (Fig. 3,d and e). If input partners are stratified within the neuropil layer, or if their innervation times differ, spatial compartmentalisation of synapses may, in part, be a result of the spatial or temporal availability of these partners during development. This allows for promiscuity during development, while maintaining the spatial compartmentalisation of synapses onto these dendrites.

### Future Analysis and Perspectives

Multiple avenues of future work open up in order to better understand the morphological structure of T4 and T5 dendrites from connectomics data. The most obvious is to investigate comparative differences in T4 and T5 between datasets, utilising the available male optic lobe connectome which has been recently released [70]. This would allow us to better capture variability across individuals, and also allow for sex-specific comparisons relating to differing size, shape, and orientation of *Drosophila* compound eyes between males and females across species [23].

Second, it is important to understand the synaptic distributions and columnar relationships in T4 and T5. Synaptic inputs are vital to dendritic development [71–73]. Further, the origin of the distinct spatial compartmentalisation of synapses onto T4 and T5 dendrites is not well understood. Extending our analysis to included synaptic, columnar, and, by extension, visual space, would prove insightful, however see [14, 74] for analysis of T4 and T5 neurons which includes visual space mapping.

### Limitations

The data presented here are from a single adult female *Drosophila*, offering a view of a single snapshot of T4 and T5 dendrite morphology. Although thought of as complete, and offering a new gold standard in data quality, some noise is undoubtedly introduced from the complex processing steps needed to generate a usable dataset from the EM image stack. We ensure that all dendrites are labelled as complete within the flywire dataset, as well as manually verifying the accuracy of dendrite annotation. However, particularly small branches are likely missing from the morphological reconstructions of neurons. We argue that as this error is likely missing at random, and the n of T4 and T5 neurons is sufficiently large, this error rate is likely uniform across the dataset and would not change any of our findings. As these data also come from a single *Drosophila*, it is also possible that different individuals show differing results. Given the fundamental nature of T4 and T5 neurons in early visual processing and motion detection, we feel this is unlikely to be the case. The question of individual variability between datasets is an intriguing one which requires further work and comparative analysis between datasets in order to be fully understood.

### Conclusions

In summary, we show that the underlying structure of T4 and T5 dendrites, as well as their subtypes, is indistinguishable. There are however, some important type- and subtype-specific differences in the manner of spatial occupancy and initial dendritic branching. With this detailed characterisation of T4 and T5 dendritic morphology, and given the observed homogeneity, we reason that the underlying developmental algorithm is likely conserved across all T4 and T5, and their subtypes. However, the mechanisms underlying these dendrites’ characteristic orientation cannot be clearly identified here.

## Materials and Methods

### Data and Code Availability

Flywire neuron ids and types are published with [21]. Specific neuron IDs used within this manuscript, as well as type and subtype annotations are available within the related github repository. The open source Python toolboxes ”fafbseg” [75], ”skeletor” [34], and ”navis” [33] were used to obtain and skeletonize neuron meshes. All further analysis and plots were generated with custom python code and toolboxes. Analysis code and datasets of all extracted morphometrics are available online (https://github.com/borstlab/T4_T5_Dendrite_Morphology_Paper).

### T4 and T5 Neuron Morphologies

The complete Flywire dataset comprises of ∼ 130000 annotated neurons, proofread and annotated by an extensive community of researchers and citizen scientists [19, 20, 29]. The optic lobe specific dataset has been published previously in [21], which provides the annotations for reconstructions used within this study. A total of 5919 T4/T5 neurons have been identified within the dataset, and 5653 individual dendrites were accurately extracted for analysis. Supplementary Table 1 gives counts by subtype for available and included dendrites. All reconstructions have been annotated as complete within the flywire dataset. After skeletonization, all neurons are converted to *μm* from *nm*, and resampled to 0.1*μm*.

### Neurons as Tree Graphs

Skeletons were extracted with the wavefront algorithm [34], and each skeleton is a tree-graph representation of a neuron. Each tree-graph is rooted at the soma, in the case of complete neurons, or the first common ancestor of nodes within the dendritic subtree (referred to as the dendrite root node), and all edges are oriented away from the root node. For completeness, we provide a brief overview of graph-theory specific to directed tree-graphs here and the components relevant to this manuscript.

A neuron is modelled as a directed tree:

(1) $$T=(V,E,r),\;r\in V\subset {\mathbb{R}}^{3},$$

where V is the set of 3-D coordinates defined by the skeletonization algorithm and E⊂V×V the set of oriented edges. r denotes the root node of the tree. e=(u→v) denotes an edge from u to v, where u is the source node, and v the target.

Each edge carries the Euclidean length $w(e)=\|v-u{\|}_{2}$. The tree weight (total cable length) is:

(2) $$W(T)=\sum_{e\in E}w(e).$$

For v∈V :

(3) $$\begin{array}{l} \deg^{+}(v)=|\{(v,w)\in E\}|,\\ \deg^{-}(v)=|\{(u,v)\in E\}|,\\ \;\deg(v)=\deg^{+}(v)+\deg^{-}(v). \end{array}$$

Where $\deg^{+}$ denotes the out-degree of a node, $\deg^{-}$ denotes the in-degree, and deg(v) the total degree. As edges point away from the root, $\deg^{-}(r)=0$, and $\deg^{-}(v)=1$ for every v≠r. branch nodes have $\deg^{+}\geq 2$ and leaf nodes $\deg^{+}=0$. An edge is *external* if $e_{v}$ is a leaf, otherwise *internal*.

For any two nodes u, v∈V there exists a unique undirected path

(4) $$P_{V}(u,v)=\left(v_{0}=u,v_{1},\ldots ,v_{k}=v\right),$$

with k=d(u,v) steps.

The corresponding edge sequence:

(5) $$\begin{array}{l} P_{E}(u,v)=\left(e_{0},e_{1},\ldots ,e_{k-1}\right),\\ \;e_{i}=\left(v_{i}\to v_{i+1}\right)\;\text{or}\;\left(v_{i+1}\to v_{i}\right), \end{array}$$

lists the k oriented edges traversed in order; note that orientation may be reversed relative to E when the walk proceeds toward the root.

Unweighted and weighted path lengths are

(6) $$\begin{array}{l} \;d(u,v)=k,\\ d_{w}(u,v)=\sum_{i=0}^{k-1}w\left(e_{i}\right). \end{array}$$

Node depth is its unweighted distance to the root, $h(v)=d_{w}(r,v)$, and an edge inherits the depth of its distal node.

Every maximal path of intermediate nodes with $\deg^{+}(v)=\deg^{-}(v)=1$ is contracted into a single edge whose weight is the sum of the collapsed weights. The reduced graph is $T_{r}=\left(V_{r},E_{r}\right)$. Such reduced trees dramatically minimise the number of nodes and edges within each neuron skeleton.

For any node v∈V we define the *subtree* rooted at v as:

(7) $$\begin{array}{l} T_{v}:=\left(V_{v},E_{v}\right),\\ V_{v}=\{x\in V|v\underset{-}{≺}x\},\\ E_{v}=\left\{(y\to z)\in E|y,z\in V_{v}\right\}, \end{array}$$

where $v\underset{-}{≺}x\;$ means that v lies on the unique root–to–x directed path in T. Thus $T_{v}$ contains v and all its descendants together with the edges that connect them, and is itself a rooted, oriented tree with root v.

### Branching Structure and Symmetry Quantification

Considering a tree graph with no nodes where $\deg^{+}=\deg^{-}=1$, as is the case for the reduced neuron skeleton graphs used here, let n=|V| and L be the number of leaves. Internal node number I=n−L. As (i) a *star* tree has one branch point and L=n−1, and (ii) a *full binary* tree satisfies L=I+1, hence I=(n−1)/2, thus for any tree graph we have an upper bound:

(8) $$1\leq I\leq \frac{n-1}{2},$$

This provides limits between maximally condensed and maximally branching tree-graphs, and no tree-graph can exist outside of these bounds. The closer I is to (n−1)/2, the closer to a full binary tree.

Tree asymmetry for a single node, $A_{v}$, as defined in [40, 41], is extended similarly to [42] in order to weight the metric by the weight of the subtree defined at v with k≥2 child nodes. In the case of $\deg^{+}(v)>2$, we use a mean of all child pairs (p,q):

(9) $$A_{v}=\frac{1}{\left(\begin{array}{c} k\\ 2 \end{array}\right)}\sum_{1\leq p<q\leq k}\left(\frac{\left|L\left(T_{p}\right)-L\left(T_{q}\right)\right|}{\left(L\left(T_{p}\right)+L\left(T_{q}\right)-1\right.}\cdot \frac{W\left(T_{p}\right)+W\left(T_{q}\right)}{W(T)}\right),$$

$A_{v}$ lies in [0, 1], where 0 indicates full symmetry in the branching structure.

### Dendrite Extraction and Data Pre-processing

We use a semi-automated method in order to annotate neuron dendrites. We first manually verify the correct rooting of each neuron tree graph at the soma. We then identify the subtree in T which maximises the number of leaves within the subtree whilst minimizing the sum of edge weights using a simple metric, Φ:

(10) $${\text{Φ}}^{\max}=\underset{v\in B}{\max}\left[\left(1-\frac{W\left(T_{v}\right)}{W(T)}\right)+\frac{L_{v}}{L}\right]$$

Where B is the set of branch nodes within T. Although often the identified subtree will be the dendrite within T4 and T5 neurons, due to either routing errors resulting from the surface mesh and skeletonization procedure, or instances where no single node isolates the root of the dendritic subtree, a further manual step is needed using a custom made GUI. Each neuron dendrite is manually verified as being the complete dendritic subtree. In cases where this is not identified accurately as described above, the reviewer has the ability to manually isolate the dendrite where possible by annotating a single chose node. Dendrites which cannot be completely isolated are excluded from analysis.

### Alignment and Scaling

In order to globally align the T4 and T5 dendrite populations, we need to define a suitable shared space for both populations. Taking the 3D point cloud of dendrite tree-graph nodes we first set the origin of the coordinate system to be the centre of a fitted sphere, separately for each population, before aligning the primary eigen axis to the polar spherical axis, or y-axis and the secondary eigen-axis with the equatorial plane, or the x-axis. Using the column assignments available in FLYWIRE, we find example dorsal, ventral, anterior and posterior columns (Fig. 1,d, insert). We then rotate the population point cloud in a manner which aligns with the Dorsal / Ventral and Anterior / Posterior axis in the column space, preserving the initial eigen-axis alignment. This results in all T4 and T5 dendrites being aligned so that the y-axis follows the Dorsal - Ventral axis and x-axis the Anterior-Posterior.

We use a similar procedure to align all dendrites of a single subtype. When aligning individual dendrites, we calculate the covariance matrix using a robust M-estimation procedure based on a Huber-type influence function. This is done in order to minimise the influence of node outliers on eigenvectors as a single long outreaching sections may impact the eigenvector orientation within single dendrites. After Eigendecomposition of the covariance matrix, eigenvectors are aligned similarly to the global procedure so the primary eigen-axis aligns with the y-axis, the secondary eigen-axis to the x-axis and the third to the z-axis. Before calculating angles of rotation we ensure that each eigenvector is oriented along it’s positive target basis vector, in order to minimise rotation.

In order to scale point node positions we scale coordinates along their Eigenbasis by s. In the case of Fig. 2.a, s is a constant defined as 1/(μ+3σ) where μ is the population mean of the highest eigenvalue $\left(\lambda_{1}\right)$ and σ the population standard deviation of $\lambda_{1}$. This scales all dendrites along each axis to $1=\mu_{\lambda_{1}}+3\sigma_{\lambda_{1}}$. Alternatively, for unit variance scaling s is a vector equal to $1/\mathrm{var}\left[e_{i}\right]$ where $\mathrm{var}\left[e_{i}\right]$ is the variance along each eigenvector, e. This scales dendrite to have equal variance along each axis.

### Surface Fitting, Volume, and Depth Measurement

In order to find the spanning volume of single dendrites, a convex hull is fit to the node coordinates of each dendrite using scipy.spatial.ConvexHull [76]. We reconstruct surfaces meshes of M10 and L1 via voxelization and marching cubes of all node coordinates in T4 (Medulla layer 10) or T5 (Lobula layer 1) dendrites. Step by step, we first compute the Axis-aligned bounding box for the given point cloud, extended by a fixed padding factor (2*μm*) to ensure a spatial margin. This is then discretized into 2*μm*^3^ voxels and each point is assigned to a voxel. 2 iterations of morphological dilation are applied in order to fill small gaps in the set of occupied voxels, and then unoccupied voxels are removed, retaining only the largest connected region of voxels. This binary grid is then converted to a continuous surface using the marching cubes algorithm as implemented in scikit-image. The resulting mesh is then cleaned by removing unreferenced nodes, duplicated or degenerate faces, filling holes in the mesh surface, and correcting face normals to ensure they are outwardly oriented. Surface meshes are smoothed using the Windowed Sinc method and a band pass filter with a width of 0.01*μm*, as implemented in [77].

In order to calculate the local depth of a point within each layer mesh we first define an inner and outer surface to the mesh, relative to the origin of the global coordinate space. This is done by sub-setting mesh face normals based on the dot product of their normals and a vector from the coordinate origin to the face centre. Faces with values ≥ 0.3 are treated as belonging to the outer mesh surface, and values ≤ −0.3 as the inner surface. A KDTree is calculated for both the inner and outer surface. For any given point, the nearest neighbour distance is calculated to both surfaces. We then calculate a normalized distance for the point from the inner surface. As T5 dendrites innervate Lobula layer 1 from the opposite side of curvature in comparison to T4 dendrites innervating Medula layer 10, we invert T5 depths. Depth is calculated as follows:

(11) $$d_{norm}=\left\{\begin{array}{cc} \frac{d_{inner}}{d_{inner}+d_{outer}} & ,T4\\ 1-\frac{d_{inner}}{d_{inner}+d_{outer}} & ,T5 \end{array}\right.$$

### Dendrite Root Nearest Neighbour Matching.

In order to identify dendrite root position nearest neighbours we first assign subtype specific nearest neighbours using KDTrees within each type - subtype combination in order to identify the distance between each T4a, for example, and the closest T4a (not including itself), T4b, T4c, and T4d dendrite root.

In order to determine the probability of a nearest neighbour being of either a specific classification (”opposite”, ”same”, or ”orthogonal”), or specific subtype, we calculate an optimal pairing within all T4 and all T5 dendrite root positions using a maximum-cardinality matching approach as implemented in [78]. This method aims to match as many points as possible, maximising the number of pairs while simultaneously minimising the total euclidean distance between the matched pairs.

### Geometry Analysis.

When calculating angles between vectors in ${\mathbb{R}}^{3}$ we project vectors $\left[v_{1},v_{2}\right]$ to the plane orthogonal to the normal vector, n, by taking the rejection of each vector from n:

(12) $$v_{i}^{⊥}=v_{i}-\frac{v_{i}\cdot n}{\|n{\|}^{2}}n$$

We then calculate the signed angle, θ, between $v_{1}$ and $v_{2}$ in the plane as:

(13) $$\theta =\mathrm{atan}2\left(\left(v_{1}^{⊥}\times v_{2}^{⊥}\right)\cdot \hat{n},v_{1}^{⊥}\cdot v_{2}^{⊥}\right)$$

Where $\hat{n}$ is the unit normal of the basis vector, $v_{1}^{⊥}\cdot v_{2}^{⊥}$ is the cosine of the angle. $\left(v_{1}^{⊥}\times v_{2}^{⊥}\right)\cdot \hat{n}$ gives the signed sine, where the sign is determined by the right hand rule with respect to n. atan2(y,x) returns the signed angle θ∈(−π,π].

As defined in [79], the circular mean is calculated as:

(14) $$\bar{\theta }=\left(\sum_{i=1}^{n}\cos\;\theta_{i},\sum_{i=1}^{n}\sin\;\theta_{i}\right).$$

and Variance as:

(15) $$V=\sqrt{{\left(\sum_{i=1}^{n}\cos\;\theta_{i}\right)}^{2}+{\left(\sum_{i=1}^{n}\sin\;\theta_{i}\right)}^{2}}.$$

The Dihedral angle β, is the ”the angle between the daughters’ half-plane and the plane formed by the parent segment and the line perpendicular to daughters’ bisection through the bifurcation point in the daughters’ plane” [37] (Fig. 5,l, insert). In order to calculate this for a set of parent and two child vectors $\left[p,c_{1},c_{2}\right]$ centred on their branching point, we first find the bisector, b, of $c_{1}$ and $c_{2}$ as:

(16) $$b=\frac{v_{1}/\left\|v_{1}\right\|+v_{2}/\left\|v_{2}\right\|}{\left\|v_{1}/\right\|v_{1}\left\|+v_{2}/\right\|v_{2}\|\|}.$$

We then calculate the angle between b and p from the perspective of the plane defined by the normal vector, n, given by:

(17) $$n=\left(c_{1}\times c_{2}\right)\times b$$

This results in the Dihedral angle β which is between 0 and π. At both extremes, this value represents bifurcations sitting on the same plane, however at 0 child vectors are on the plane oriented in the same direction as the parent relative to the bifurcation points, and at π they will be oriented away.

In order to generate bifurcation random null-models we randomly generate 1, 000, 000 sets of three 3-dimensional unit vectors. We ensure that one vector is oriented towards the negative x-axis to represent the parent section, and the other two are oriented along the positive x-axis. This ensures an approximation of the outward facing branching structure observed. Second, sets of bifurcation vectors are then scaled along their z-axis separately for T4 and T5 by the cube root of the mean variance explained by the third principle component, in order to mimic the natural flatness of the occupied space of T4 and T5 dendrites.

### Explorative Distribution Modelling

We divide dendrite populations by type (T4, T5), subtype (a,b,c,d) and section type (internal, external). We then fit using maximum likelihood, for each group, six test distributions in order to compare goodness of fit. The distributions used are as follows:

Wald (Inverse Gaussian) Distribution, with mean μ>0 and shape λ>0:

(18) $$f(x|\mu ,\lambda )=\sqrt{\frac{\lambda }{2\pi x^{3}}}\exp\left(-\frac{\lambda {\left(x-\mu \right)}^{2}}{2\mu^{2}x}\right),x>0.$$

Log-normal Distribution, with log-mean μ∈ℝ and log-standard deviation σ>0:

(19) $$f(x|\mu ,\sigma )=\frac{1}{x\sigma \sqrt{2\pi }}\exp\left(-\frac{{\left(\ln x-\mu \right)}^{2}}{2\sigma^{2}}\right),x>0.$$

Gamma Distribution, with shape k>0 and scale θ>0:

(20) $$f(x|k,\theta )=\frac{x^{k-1}e^{-x/\theta }}{\text{Γ}(k)\theta^{k}},x>0.$$

where Γ is the gamma function.

Exponential Distribution (Gamma distribution special case with k=1) with scale θ>0:

(21) $$f(x|\theta )=\frac{1}{\theta }e^{-x/\theta },x\geq 0.$$

Log-logistic Distribution with shape c>0 and scale s>0:

(22) $$f(x|c,s)=\frac{c}{s}{\left(\frac{x}{s}\right)}^{c-1}{\left(1+{\left(\frac{x}{s}\right)}^{c}\right)}^{-2},x>0.$$

Minimum Weibull Distribution with shape c>0 and scale λ>0:

(23) $$f(x|c,\lambda )=\frac{c}{\lambda }{\left(\frac{x}{\lambda }\right)}^{c-1}\exp\left[-{\left(\frac{x}{\lambda }\right)}^{c}\right],x\geq 0$$

For each fitted distribution we calculate the Bayesian Information Criterion (BIC) as:

(24) $$\text{BIC}=k\;\ln\;n-2\ell (\hat{\theta })$$

where k is the number of free parameters (2 for Wald, log-normal, gamma, log-logistic, and Weibull Distributions; 1 for exponential). n is the number of observations within the sample and $\ell (\hat{\theta })$ is the maximised log-likelihood. Lower BIC indicates a better trade off between model fit and parsimony.

### Statistical Analysis

We test for type (T4, T5) and subtype (a,b,c, and d) effects on different measured variables using ordinary least squares (OLS) regression and analysis of variance (ANOVA) / covariance (ANCOVA). Models are fit with a full factorial structure, and sum of squares were evaluated using Type III tests. We additionally, for each effect (main effects and interaction terms), calculate the partial eta-squared $\left(\eta_{p}^{2}\right)$ as an effect size:

(25) $$\eta_{p}^{2}=\frac{SS_{effect}}{SS_{effect}+SS_{error}}$$

where SS is the sum of squares. We additionally report slopes (β) obtained from the regression model fit for meaningfully large main effects, as well as group specific slopes and their differences (Δβ) where applicable.

We additionally obtain, for each effect size, a non-parametric confidence interval obtained through stratified bootstrap resampling (1000 samples). *Post hoc* pairwise comparisons were conducted on marginal means, both at the main effect level and within interactions, and Cohen’s d was calculated for effect sizes:

(26) $${\text{Cohen’s}}_{d}=\frac{\mu_{i}-\mu_{j}}{s_{pooled}}$$

with

(27) $$s_{pooled}=\sqrt{\frac{\sigma_{i}^{2}+\sigma_{j}^{2}}{2}}$$

where μ is the group mean and σ the group standard deviation. Again, non-parametric confidence intervals using 1000 bootstrap resamples are calculated.

### Plotting Conventions.

We make liberal use of the empirical probability mass functions (epmf) for plotting distributions throughout, without smoothing. To allow for comparison within plots, binning along the x-axis is the same for all epmf’s shown within a plot. Bin counts and ranges are adjusted manually. Error bars are presented in two forms, as specified in a plot by plot basis. In cases where a single observation is available per individual dendrite, a bootstrapped confidence interval around the mean in each bin is shown (1000 bootstrap replicates). Alternatively, when a repeated measure is available within each dendrite, a single epmf is generated for each individual dendrite and, in order to give an idea of individual variability across the population, error bars within each bin ar calculated as the asymmetric median absolute deviation (MAD_*a*_:

(28) $${\text{MAD}}_{\text{low }}=\mathrm{median}\left(\left|x_{i}-\tilde{x}\right||x_{i}<\tilde{x}\right)\;{\mathrm{MAD}}_{\text{high }}=\mathrm{median}\left(\left|x_{i}-\tilde{x}\right||x_{i}>\tilde{x}\right)$$

where $\tilde{x}$ is the median within the respective bin. This illustrate variance in each bin across neurons. An Asymmetric MAD allows for the possibility that individual variability within each bin is skewed, and can be shown within the plot.

## Supplementary Material

Supplement 1 **S1 Fig. Supplementary Figure 1**: Distribution and Correlations of Size Based Dendrite Metrics. a) Probability mass function of total dendrite cable lengths. b) Spatial distribution of total dendrite cable length. c) Correlation between total dendrite cable length and dendrite convex hull volume. d) Probability mass function of total number of dendrite sections. e) Spatial distribution of total number of dendrite sections. f) Correlation between total number of dendrite sections and convex hull volume.
**S1 Table.** Counts of all available and included neuron morphologies before and after dendrite annotation.
