# Supplementary material for: Population Morphology Implies a Common Developmental Blueprint for Drosophila Motion Detectors
Source: bioRxiv. 2025 Dec 21:2025.11.15.688637. Preprint. [Version 3] doi: 10.1101/2025.11.15.688637 (PMC12724164; doi:10.1101/2025.11.15.688637)
Supplement: Supplement 1 — S1 Fig. Supplementary Figure 1: Distribution and Correlations of Size Based Dendrite Metrics. a) Probability mass function of total dendrite cable lengths. b) Spatial distribution of total dendrite cable length. c) Correlation between total dendrite cable length and dendrite convex hull volume. d) Probability mass function of total number of dendrite sections. e) Spatial distribution of total number of dendrite sections. f) Correlation between total number of dendrite sections and convex hull volume. S1 Table. Counts of all available and included neuron morphologies before and after dendrite annotation. [file media-1.pdf]

Supplementary Material

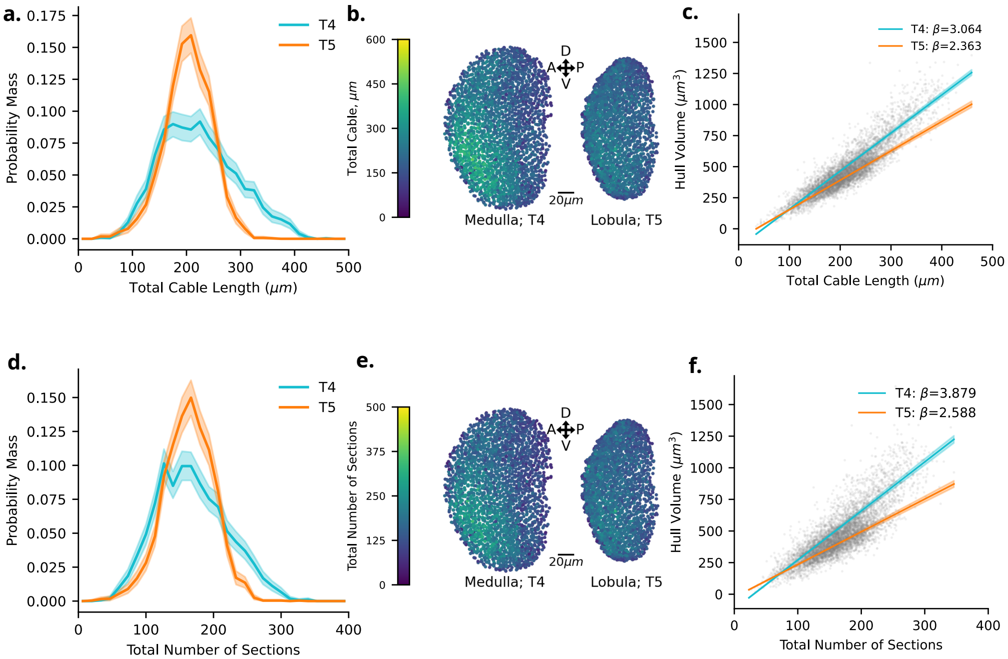

Fig 1. Supplementary Figure 1: Distribution and Correlations of Size Based Dendrite Metrics. a) Probability mass function of total dendrite cable lengths. b) Spatial distribution of total dendrite cable length. c) Correlation between total dendrite cable length and dendrite convex hull volume. d) Probability mass function of total number of dendrite sections. e) Spatial distribution of total number of dendrite sections. f) Correlation between total number of dendrite sections and convex hull volume.

Supplementary Results

When comparing total dendrite cable length, we find a small main effect of Type ($\eta_p^2 = 0.024, \mathrm{CI} : [0.017, 0.032]$). *Post-hoc* pairwise comparisons reveal that the mean total cable length of T4 dendrites is greater than T5 with a small effect size ($\mathrm{Cohen's}_d = 0.327, \mathrm{CI} : [0.281, 0.373]$, Fig. S1, a). When considering total number of sections, we find no meaningfully large effects of Type, Subtypes, or Type:Subtype interaction (Fig. S1, d). Considering the spatial distribution of total cable and total sections, and the tail of T4 distributions, as in Fig. 2,f-g we observe the same spatial organisation showing larger dendrites within the ventral-anterior region of Medulla Layer 10 in T4, not present in T5 (Fig. S1, b and e). Treating total dendrite cable length as a covariate predictor for dendrite convex hull volume we find a large main effect for total cable length ($\eta_p^2 = 0.424, \mathrm{CI} : [0.375, 0.479]$) with a slope of $\beta = 3.118$, as

well as a small main effect for the Type:Total cable length interaction term $\eta_p^2 = 0.018, \mathrm{CI}: [0.011, 0.026]$. Using the section count as a covariate we find a similar result with a large effect size for section count ($\eta_p^2 = 0.278, \mathrm{CI}: [0.243, 0.327]$) and a small effect size for the interaction between section count and type ($\eta_p^2 = 0.027, \mathrm{CI}: [0.019, 0.037]$). In both cases, this illustrates the steeper slope within T4 than in T5 (Fig. S1, c and f).

Subtype	Count in Flywire	Count Used	Proportion Used
T4a	729	706	0.97
T4b	744	717	0.96
T4c	777	761	0.98
T4d	748	738	0.99
T5a	733	676	0.92
T5b	743	694	0.93
T5c	737	693	0.94
T5d	708	668	0.94

Table 1. Counts of dendrites by subtype used within the study and available in flywire
